# Direct observation of room-temperature out-of-plane ferroelectricity and tunneling electroresistance at the two-dimensional limit

**DOI:** 10.1038/s41467-018-05662-y

**Published:** 2018-08-20

**Authors:** H. Wang, Z. R. Liu, H. Y. Yoong, T. R. Paudel, J. X. Xiao, R. Guo, W. N. Lin, P. Yang, J. Wang, G. M. Chow, T. Venkatesan, E. Y. Tsymbal, H. Tian, J. S. Chen

**Affiliations:** 1 Department of Materials Science and Engineering, National University of Singapore, Singapore, 117575 Singapore; 2 Center of Electron Microscope, State Key Laboratory of Silicon Materials, School of Materials Science and Engineering, Zhejiang University, 310027 Hangzhou, China; 3 Department of Physics and Astronomy & Nebraska Center for Materials and Nanoscience, University of Nebraska, Lincoln, NE 68588-0299 USA; 4 Singapore Synchrotron Light Source (SSLS), National University of Singapore, Singapore, 117603 Singapore; 5 NUSNNI-Nanocore, National University of Singapore, Singapore, 117411 Singapore

## Abstract

Out-of-plane ferroelectricity with a high transition temperature in nanometer-scale films is required to miniaturize electronic devices. Direct visualization of stable ferroelectric polarization and its switching behavior in atomically thick films is critical for achieving this goal. Here, ferroelectric order at room temperature in the two-dimensional limit is demonstrated in tetragonal BiFeO_3_ ultrathin films. Using aberration-corrected scanning transmission electron microscopy, we directly observed robust out-of-plane spontaneous polarization in one-unit-cell-thick BiFeO_3_ films. High-resolution piezoresponse force microscopy measurements show that the polarization is stable and switchable, whereas a tunneling electroresistance effect of up to 370% is achieved in BiFeO_3_ films. Based on first-principles calculations and Kelvin probe force microscopy measurements, we explain the mechanism of polarization stabilization by the ionic displacements in oxide electrode and the surface charges. Our results indicate that critical thickness for ferroelectricity in the BiFeO_3_ film is virtually absent, making it a promising candidate for high-density nonvolatile memories.

## Abstract (web summary)

High temperature perpendicular ferroelectricity in nano thin films is crucial for miniaturization of electronic devices. Here the authors show the presence of stable and switchable out-of-plane ferroelectricity in tetragonal BiFeO_3_ thin films at the two-dimensional limit and 370% tunneling electroresistance in ferroelectric tunnel junctions.

## Introduction

In condensed matter, order parameters, such as ferromagnetic magnetization^1^, superconducting energy gap^2^, and ferroelectric polarization^3–5^, are usually suppressed at reduced dimensions due to size, surface, and interface effects, and quantum fluctuations^6^. Recently, it has been observed that a monolayer Fe film exhibits a long-range ferromagnetic order^7^ and two-dimensional superconductivity exists in a single layer metal film^8^ and an FeSe film^9^, which entirely break the dimensional limit of long-range order. In ferroelectricity, although robust in-plane spontaneous polarization has been experimentally observed in atomic-thick SnTe^10^, it is considered that there exists a critical thickness for out-of-plane ferroelectricity in a pristine film due to an intrinsic depolarizing field, arising from incomplete screening of polarization charges at the ferroelectric (FE)/metal interface^11^, and the extrinsic effects of interfacial strain^12^, misfit dislocations^13^, and surface reconstruction^14^. Theoretically, the critical thickness of 2.4 nm has been predicted for BaTiO_3_ (BTO)^15^, whereas experimentally it has been shown that there exists a critical thicknesses of 1.2 nm for PbTiO_3_ (PTO)^16^ and 1 nm for copolymer^17^. Recently, spontaneous polarization with 17% of the bulk value has been observed in 1.5-unit cells (u.c.) thick PbZr_0.2_Ti_0.8_O_3_ (PZT) film thinned by ion milling^18^. So far, however, direct growth of atomically thick FE films with polarization normal to the film surface at room temperature remains a challenge even though desired for high-density nanodevices.

Ferroelectricity in ultrathin films has mainly been characterized by Raman spectroscopy^5^, X-ray scattering^16^, X-ray photoelectron diffraction^19^, and scanning transmission electron microscopy (STEM)^18^. The presence of stable and switchable polarization in an FE film at the two-dimensional limit has not been verified and directly observed yet. To reveal ferroelectricity in an ultrathin film, local atomic-scale information on the atomic displacement is needed in conjunction with the mesoscopic polarization switching. BiFeO_3_ (BFO), a lead-free multiferroic material, is promising for non-volatile memories and electrically controlled magnetism due to its significant remnant polarization and magnetoelectric coupling above room temperature^20^. Even though ferroelectric polarization of BFO could be enhanced by surface boundary conditions^21^, ferroelectricity vanishes below 2 nm thickness^22^, limiting its practical application in miniaturized electronic devices.

In this work, we report coherent growth and room temperature stable ferroelectricity of BFO films on the tetragonal SrRuO_3_ (SRO)-buffered STO (001) substrate. Using aberration-corrected STEM images and energy dispersive X-ray (EDX) maps, we visualize, at the atomic level, the out-of-plane atomic displacement in the two-dimensional BFO films. The existence of switchable and stable polarization is verified using piezoresponse force microscopy (PFM), while the switching mechanism is investigated using Kelvin probe force microscopy (KPFM) and the polarization stability is explained based on the first-principles calculations. A surprisingly high-tunneling electroresistance effect of ~370% is observed in ferroelectric tunnel junctions (FTJ) using a 1-u.c.-thick BFO film as a barrier at room temperature.

## Results

### Direct observation of spontaneous polarization at the two-dimensional limit

**Fig. 1:**
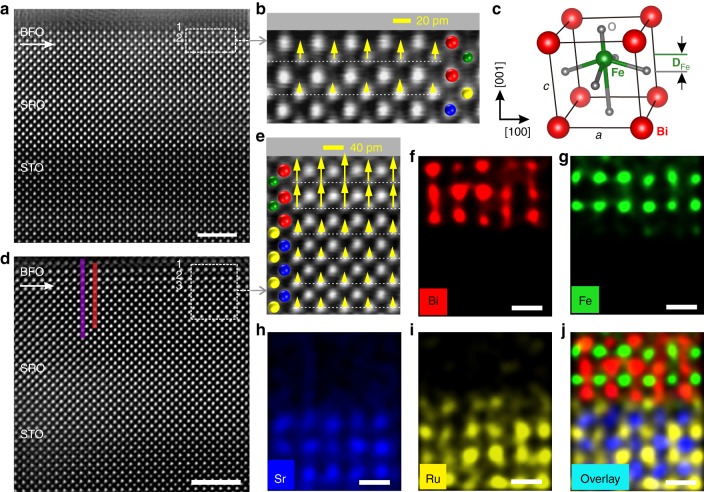
Atomic-scale observation of polarization in 2- and 3-u.c. BFO. **a** Atomic-resolution HAADF-STEM image of 2-u.c. BFO. The scale bar is 2 nm. **b** Superposition of a magnified image and B-site atomic displacement vector map from the area marked with a white dashed rectangle in **a**. **c** Schematic of the unit cell of ferroelectric BFO, **D**_Fe_ denotes the relative displacement of the Fe atoms. **d** HAADF-STEM image of 3-u.c. BFO film. The scale bar is 2 nm. **e** Superposition of a magnified image and B-site atomic displacement vector map from the area marked with a white dashed rectangle in **d**. In the insets of **b**, **e**, yellow arrows represent the displacement vectors of B-site atoms and white dashed lines are drawn to guide visually. The length and direction of the arrows represent the magnitude and direction of the displacement vectors concerning the scale bar in the upper right corner, respectively. **f**–**j** The false-color Bi, Fe, Sr, Ru, and overlaid EDX elemental maps of **e**, respectively. The scale bar is 0.5 nm

The ferroelectric polarization and interfacial chemical environment are directly determined at the atomic level by aberration-corrected STEM^12,23^. Figure 1 and Supplementary Figure 2 show the typical STEM images of the cross-section along the [001] zone axis. Atomically resolution high-angle annular dark-field STEM (HAADF-STEM) images illustrated in Fig. 1a, d reveal that both BFO and SRO are coherently grown on the STO substrate. As indicated by the clear contrast from the HAADF images, SRO/BFO and SRO/STO interfaces are sharp, consistent with the AFM and XRD results (Supplementary Figures 1 and 27). Atomic-resolution energy dispersive X-ray (EDX) maps of 3-u.c. BFO are presented in Fig. 1f–j. The number of unit cells in BFO is clearly seen from the positions of Fe and Bi atoms. The interfacial terminations between BFO and SRO are identified as –RuO_2_–BiO–, which is different from the previous works^22,24^. Using two-dimensional (2D) Gaussian fitting of the STEM image intensity, displacement vector (**D**_Fe_) maps of Fe atoms with respect to the mass center of the Bi sublattice (as shown in Fig. 1c) are determined unit cell by unit cell (details in Supplementary Note 5), shown in Fig. 1b, e. The outer unit cell of BFO is not considered to avoid the possible effect on the displacement vector from deposited Pt layer in the process of focused ion beam sample preparation. The average value of **D**_Fe_ in 2-u.c. and 3-u.c. BFO is about 19 pm (15–25 pm) and 35 pm (30–50 pm), respectively. Figure 1b, e reveals a monodomain polar phase in both 2-u.c. and 3-u.c. BFO films, which is different from a 180° strip domain structure usually formed in ultrathin PTO on STO^16^. This difference suggests that the depolarizing field is effectively compensated by free charges from tetragonal SRO electrode, not like in the case of PZT (20 nm)/SRO (3 u.c.), in which the depolarizing field is not fully screened by such a thin oxide electrode, resulting in the PZT layer having a 180° strip domain^25^.

Using the empirical linear relation (**P**_s_ = κ*D_Fe_) between the spontaneous polarization **P**_s_ and the displacement vector **D**_Fe_, the spontaneous polarization can be roughly evaluated^12^. Here *κ* is a constant of 2.50–2.58 μCcm^−2^ pm^−1^ adopted in the estimation of **P**_s_ for BTO, PTO, and BFO systems. The spontaneous polarization of 2-u.c. and 3-u.c. BFO films are estimated to be about 49 μCcm^−2^ and 88 μCcm^−2^, respectively. The polarization of 2-u.c. BFO is comparable to the reported result of thick BFO film on SRO electrodes^26^. We also examine the ferroelectric polarization at the ultimate thickness limit of a monolayer. One could observe an obvious atomic relative displacement (25–40 pm) in 1-u.c.-thick BFO films (Supplementary Figure 3). To verify the contribution of electrical boundary conditions to spontaneous polarization in ultrathin BFO films, we examine the atomic displacement of BFO in a symmetric environment (SRO/BFO/SRO) using STEM. The displacement vector map (Supplementary Figure 4) shows that the BFO films placed in a symmetric environment have a smaller atomic displacement than that in an asymmetric environment. This suppression of spontaneous polarization is also observed in SRO/BTO/SRO system, which is associated with the different depolarizing field in the two cases^27^. In light of the influence of termination of SRO electrode on the ferroelectricity, we prepared the BFO film buffered by the SRO electrode with SrO termination (details in Supplementary Note 2). We find that the atomic displacement of BFO on SrO-terminated SRO is about 25 pm, which is smaller than that of BFO on RuO_2_-terminated SRO (Supplementary Figure 5).

It is worth noting that the **D**_Fe_ of the interfacial unit cell is even larger than that of the second unit cell in some local areas of the ultrathin film, which might be caused by defects from film growth and preparation of TEM samples. To examine the uniformity of the polar displacement, we measured more samples and different regions across a larger length scale. We found all BFO films exhibiting the atomic displacement with the same orientation but slightly different absolute values (Supplementary Figure 6). These results directly show the presence of robust out-of-plane FE polarizations at room temperature in the two-dimensional as-grown BFO films. Below we demonstrate that this polarization is switchable by an applied electric field, thus revealing the ferroelectric nature of the polar state.

### Stabilizing mechanism of ferroic order

**Fig. 2:**
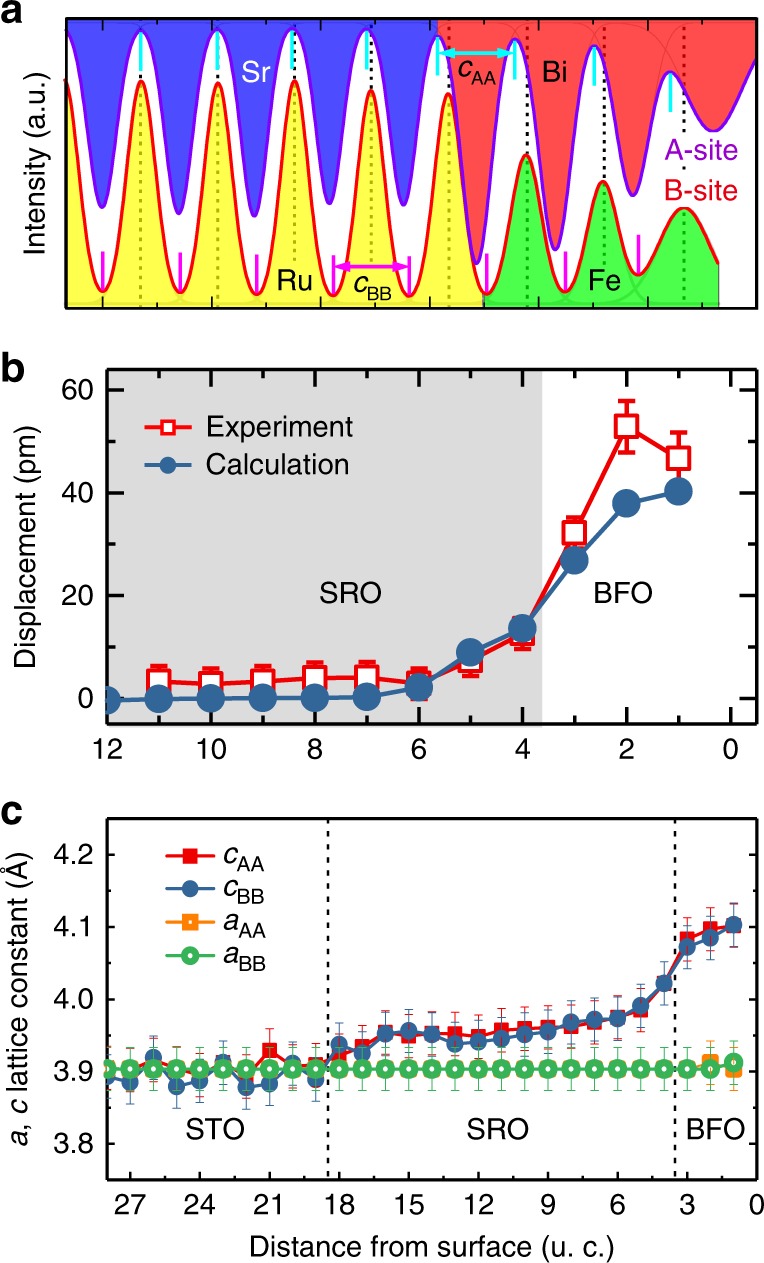
Quantitative analysis of the off-center displacement and lattice parameters in BFO by experimental and calculated data. **a** The STEM intensity profiles of A-site (top, purple) and B-site (down, red) cations across heterointerface of SRO/BFO along the purple and red line in Fig. 1d. The short cyan lines represent the center of A-site atoms, while the black dashed lines represent the position of B-site atoms. **b** The averaged off-center displacement of B-site cations nearby the SRO/BFO interface in experiment and calculation. The mean value was calculated by averaging the position value over 28-u.c. parallel to the interface from STEM images at several different areas. The simulation cell in the calculations is SRO/BFO/vacuum. The SRO region is shaded. **c** The *c* and *a* axis lattice parameters deduced from the distance of A–A (Bi–Bi, Sr–Sr) and the distance of B–B (Fe–Fe, Ru–Ru). The error bars mark the standard deviation with respect to averaging along each lattice layer

To clarify the screening mechanism of the depolarizing field at the two-dimensional limit, a quantitative analysis of atomic displacement at the interface of SRO/BFO was conducted. Figure 2a displays the STEM intensity profiles of A-site and B-site cations across the SRO/BFO heterointerface, clearly indicating a relative displacement between the B-site cation and the center of mass for A-site sublattice. The line profiles of the displacement along the growth direction in Fig. 2b indicate the relative displacement of 30–50 pm in BFO. It is notable that Ru cations in the bulk centrosymmetric SRO have a displacement ~10 pm over a distance of 2–3 u.c. from the interface. This result indicates that ionic polar displacements from the ferroelectric layer penetrate into the conductive oxide electrode, which is consistent with the theoretical predictions for PTO/SRO^25^ and BTO/SRO^28^ heterostructures. Our results show that only 1–2 u.c. of SrO-terminated SRO electrode are weakly polarized to screen a depolarizing field in. To reveal the origin of the polarized SRO, we conducted the cross-sectional STEM imaging of SRO layer without a BFO layer. We found that the tetragonality of SRO film was about 1.02–1.03, whereas the atomic displacement in SRO was less than 5 pm (Supplementary Figure 7), which agrees with the centrosymmetric structure of bulk SRO. Thus, the large off-center displacement of Ru cations near the SRO/BFO interface is caused by the polarization of BFO films.

Our first-principles calculations in Fig. 2b predict a similar trend for the displacement of B-site cations at the interface of the FE layer/oxide electrode. Theoretically, in both asymmetric and symmetric environments, ultrathin BFO films should have a similar relative displacement (Supplementary Figure 8). For the SRO/BFO/vacuum heterostructure, surface ionic charges and screening charges assist to stabilize two polarization states. The tetragonal BFO with *c*/*a* of 1.05 observed in the experiment gains an energy ~0.54 eV per unit cell with respect to the paraelectric phase due to ionic off-centering and volume enlargement (Supplementary Figure 9).

**Fig. 3:**
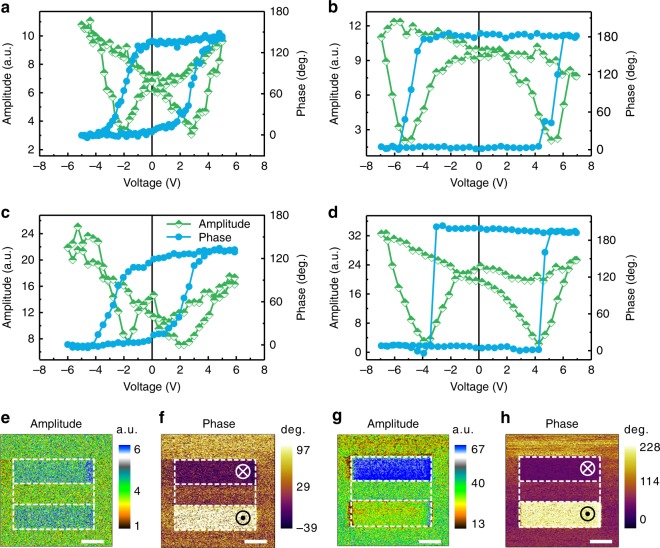
Ferroelectric switching and stability in 2- and 3-u.c. BFO films. **a**, **b** Out-of-plane SS-PFM amplitude (half-filled green squares) and phase (filled azure circles) curves for 2-u.c. BFO film measured in air and argon, respectively. **c**, **d** Out-of-plane SS-PFM amplitude (half-filled green squares) and phase (filled azure circles) curves for 3-u.c. BFO film measured in air and argon, respectively. **e**, **f** Out-of-plane PFM amplitude and phase images for 2-u.c. BFO film measured in air after applying +5 V and −5 V voltage. **g**, **h** Out-of-plane PFM amplitude and phase images for 2-u.c. BFO film measured in argon after applying +5 V and −5 V voltage. The scale bar is 1 μm

We also observe that the out-of-plane lattice parameter *c* estimated from A–A site distance and B–B site distance maintains a constant value in STO but gradually increases in SRO when approaching the SRO/BFO interface, eventually reaching the value of BFO (Fig. 3c). The in-plane lattice parameter *a* across the entire heterostructure maintains a constant value of about 0.39 nm, which reveals good coherent epitaxy. The tetragonality value (*c*/*a*) of BFO is estimated to be 1.04–1.05, which is quite close to the value measured by XRD. The strain across the SRO/BFO interface is only 0.88% (Supplementary Figure 10), suggesting not be the main factor responsible for ferroelectricity in the ultrathin BFO films. Thus, our experimental and theoretical results demonstrate that a sizable ionic polarization of the oxide electrode at the interface may be one of the reasons resulting in the critical thickness for ferroelectricity to be reduced or absent in our BFO films^28^. Note that another important reason of stabilizing ferroelectricity in the two-dimensional BFO includes screening of polarization charge at the top surface by various ionized surface adsorbates such as hydroxyl groups and protons, derived from H_2_O molecules and organic molecules^27,29,30^. The role of surface screening charges in the ferroelectric polarization switching is demonstrated below.

### FE polarization switching at room temperature

The stability and switchability of the FE polarization of two-dimensional BFO film at a larger scale were examined by high-resolution PFM. Figure 3a, c shows the out-of-plane switching spectroscopy PFM (SS-PFM) results for 2-u.c. and 3-u.c. BFO films measured in air, respectively, while Fig. 3b, d shows the SS-PFM results for the same films measured in dry high-purity argon, respectively. The in-plane PFM signals of such thin ferroelectric films are too weak to be measured in our scanning probe microscope (not shown here). The strong hysteresis behavior and butterfly-like shape are observed in phase and amplitude signals, respectively, confirming that ferroelectricity is sustained when the thickness of BFO films is down to 2-u.c. Similar behavior has been observed in other ultrathin ferroelectric films, such as 9-u.c. PTO^31^, 4-u.c. BTO^32^, and 1–2 layer PVDF^33^. Even when the BFO thickness is decreased to 1-u.c., clear hysteresis loops and butterfly-like shapes are observed (Supplementary Figure 11). Moreover, SS-PFM results show a squarer phase loop and larger coercivity when measured under dry and inert Ar atmosphere as compared to those measured in air. This is likely due to organic molecules and moisture present in air, aiding the polarization switching via domain nucleation mechanism^34,35^. On the other hand, polarization switching in Ar atmosphere occurs more coherently via continuous mechanism^35^, which results in a larger coercivity. A larger coercive voltage was also observed in our 2-u.c. sample as compared to the 3-u.c. sample. This increase in coercive voltage due to the change in the mechanism of polarization switching from domain nucleation to continuous is further confirmed by SS-PFM measurements on a 12 nm thick BFO sample (Supplementary Figure 12). The out-of-plane PFM images of 2-u.c. BFO in air (Fig. 3e, f) and in Ar (Fig. 3g, h) are consistent with the results of SS-PFM measurements. The phase and amplitude images measured in Ar show better contrast than those measured in air, which is likely associated with the surface screening charges from adsorbates. SS-PFM maps (Supplementary Figure 13) on 2-u.c. and 3-u.c. BFO under Ar atmosphere, respectively, indicate that the ferroelectric properties of BFO films are uniform over a large area.

With decreasing ferroelectric film thickness, especially for several unit cells thick film, surface screening charges considerably affect the PFM results for ultrathin BFO (Fig. 3), making them similar to those for 1-nm BTO^36^, 2-nm La-doped BiMnO_3_^37^, and 3-nm Sm-doped BFO^38^. We employ a grounded conductive tip without electrical bias to scan the bare surface of ferroelectric film (details in Supplementary Note 3), reducing the effect of such surface screening charges through a friction process^39^. A better phase contrast (Supplementary Figure 14) is obtained, which agrees with the STEM observation. Surface potential distribution after electrical poling was also measured by KPFM to understand the screening mechanism in polarization switching (Supplementary Figure 15). A change of the depolarizing field arising from the polarization switching is completely compensated by the surface charges, which assists in stabilizing the oppositely polarized state of ultrathin BFO in the electrical switching process. Time-dependent PFM signals (Supplementary Figure 16) show that the amplitude difference of poling regions decreases by few (10%) percent in the first 2 h after electrical poling, and then almost remains constant over next 48 h. It is much larger than the lifetime (<0.5 h) of non-ferroelectric signals^40^, which reveals the polar states in the two-dimensional BFO films are switchable and stable.

Note that some non-ferroelectric mechanisms, such as surface and interface-trapped charges and field-induced-ion redistribution^41,42^ can also contribute to PFM signals. To rule out those mechanisms, we employ special PFM approaches^32,42^, where PFM images are obtained after applying different *V*_DC_ voltages (Supplementary Figure 17), and local hysteresis curves are measured using different *V*_AC_ voltages (Supplementary Figures 18 and 19). Long lifetime, sharp phase contrast, and *V*_AC_ dependence of loops reveal the signature features typical for ferroelectric materials. We note that PFM measurements of SRO electrode show no ferroelectric behavior (Supplementary Figure 20). We also find the negligible PFM signals for 2-u.c. BTO and STO (Supplementary Figures 21–23). On the other hand, ferroelectricity of the ultrathin BFO films is confirmed by measuring the ferroelectric hysteresis loops directly on the top electrode (Supplementary Figure 24). Overall, combining systematic PFM measurements with KPFM characterization, we demonstrate that the out-of-plane FE polarization of ultrathin BFO film is stable and switchable, even down to a thickness of 1-u.c.

### Ferroelectric tunnel junctions with a two-dimensional BFO barrier

**Fig. 4:**
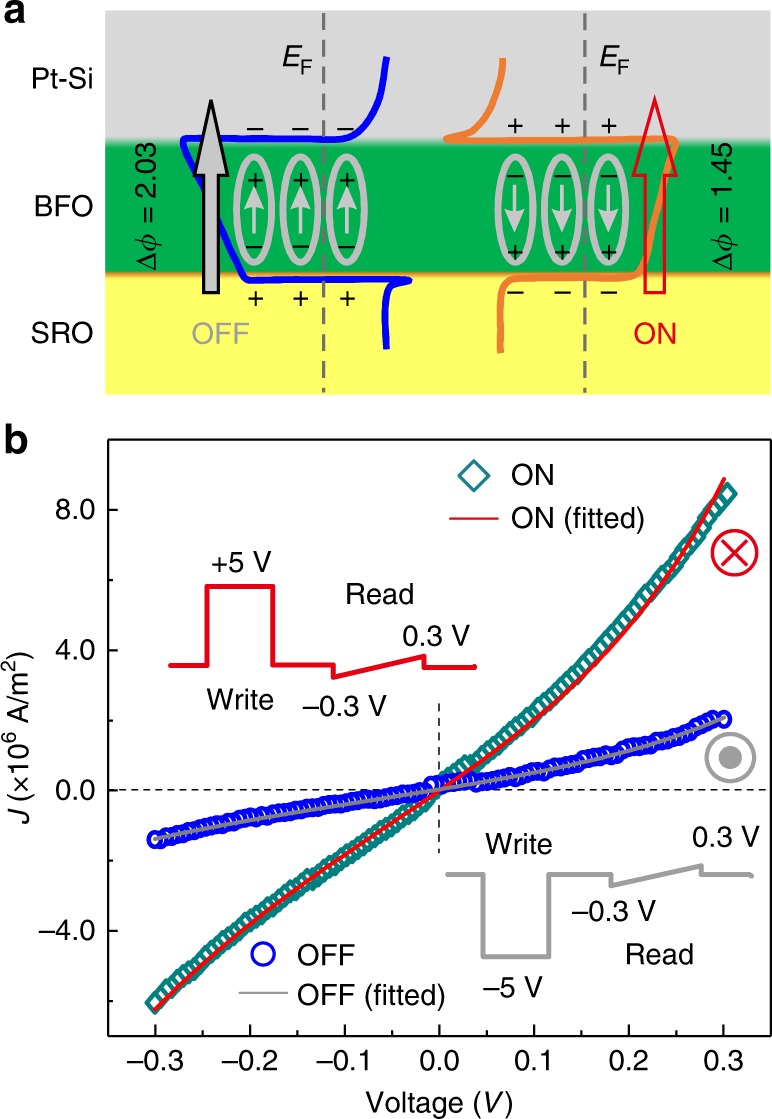
Ferroelectric tunneling junction and tunneling mechanism in 1-u.c. BFO. **a** Schematic of electrostatic potential (*ϕ*) profiles in the OFF and ON states. The Fermi level is marked by the gray dashed lines. The small arrows represent the ferroelectric polarization vector. The large solid (gray) and open (red) arrows indicate the magnitude of tunneling current. **b** *J*–*V* curves in the small range of voltage for two opposite polarization orientation (blue, down, orange, up). Solid lines show fitting of experimental data using direct tunneling mechanism based on the WKB theory. The inset in (**b**) illustrates the write and read voltage sequences for *J*–*V* measurements

Using the out-of-plane polarization of a ferroelectric ultrathin film, FTJ device with 1-u.c.-thick BFO barrier layer (Fig. 4a) was examined with conductive AFM. Polarization switching produces a high resistance (OFF) state and a low resistance (ON) state, through modulating a potential barrier height. Figure 4b shows the performance of the 1-u.c. BFO-FTJ at room temperature. Current density–voltage (*J–V*) curves between −0.3 and 0.3 V are measured after a 30 ms square write voltage pulse of ±5 V with the write and read voltage sequence (Inset of Fig. 4b) and reveal a nonlinear characteristic and a noticeable difference in resistance. Using direct tunneling equation based on the Wentzel−Kramers−Brillouin (WKB) theory extended by Gruverman et al.^43^, we could simultaneously fit the ON/OFF state conductance curves, indicating that the tunneling electroresistance (TER) effect in ultrathin BFO originates from polarization-modulated tunnel transmission^44^. To rule out the extrinsic effect from an electrode on TER, we examined transport behavior and ferroelectricity of SRO (Supplementary Figure 20). As expected, SRO electrode shows no ferroelectric response, and the *J–V* curves of SRO at the virgin state and poled state by voltage pulses exhibit a typical Ohmic resistance behavior of a metallic material. We obtain a large TER ratio (defined as TER = (*J*_on_−*J*_off_)/*J*_off_) of 370% for 1-u.c. BFO-FTJ and an even larger TER ration of 2700% for 2-u.c. BFO-FTJ (Supplementary Figure 25). We compare the performance of our tetragonal BFO (T-BFO)-FTJ with other similar ultrathin FTJs reported recently, listed in the Supplementary Table II. It is evident that the tetragonal BFO junctions display higher TER at a smaller thickness, which is very crucial for the application of FTJs in miniaturized electronic devices.

## Discussion

**Fig. 5:**
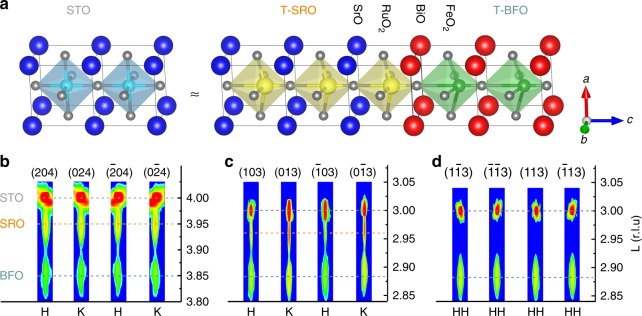
Confirmation of tetragonal structure of SRO/BFO. **a** Schematic of tetragonal BFO on tetragonal SRO buffered (001) STO. **b**–**d** In-plane asymmetric reciprocal space mappings (RSM) around the {204} (**b**), {103} (**c**), and {113} (**d**), respectively. Bragg reflection is measured on SRO (7 nm)/BFO (12 nm) heterostructures

It has been reported that ferroelectricity of BFO grown on SRO disappears below 2 nm of thickness. In those reports, the BFO films grown on the monoclinic (*P*_bnm_) SRO electrode were in the rhombohedral or monoclinic phase^22,26^. On the contrary, both BFO and SRO films in our work show coherent epitaxial growth with the STO substrate and are of purely tetragonal phase with the same orientation, as shown in Fig. 5 and supplementary Fig. S26. Also, the absence of peak-splitting in all BFO reflections reveals a single domain structure of the BFO film, which fully agrees with the STEM and PFM results. The lattice parameters were further refined via the reciprocal space vector (RSV) method (details in Supplementary Note 4) and summarized in Supplementary Table I. The *c*/*a* ratio of current BFO film is 1.04, which is larger than 1.02 of rhombohedral BFO. To further gain insight on the preservation of ferroelectricity at the two-dimensional limit, we compared our BFO with other conventional tetragonal ferroelectric materials (i.e., PZT, PTO, and BTO)^15,16^ grown on SRO electrode; there are two key differences. First, the termination of SRO at our SRO/BFO interface is B-site (RuO_2_) as shown schematically in Fig. 5a and confirmed by HAADF-STEM images. Therefore, the first FeO_2_ layer has the same neighboring chemical environment as bulk BFO and the B-site termination more effectively screens the depolarizing field than the A-site termination (Supplementary Figure 5). It has been pointed out that environment of B sites plays a key role in reducing the critical thickness of PTO for ferroelectricity^16^. Second, the tetragonality of our BFO still remains as high as 1.04 even the thickness is reduced to 1- and 2-u.c. This is in contrast to tetragonality of PTO decreasing from 1.06 to 1.01 when the thickness is reduced from bulk to 1-u.c.^3,16,19^. Only when the tetragonality is larger than 1.03 (corresponding to 3-u.c.), the ferroelectricity of PTO persists.

Recently, ferroelectricity was observed at 1.5-u.c. PZT thinned by an ion milling process, which is mainly attributed to the strong Pb–O bond or the proximity effect of thicker FE region^18^. In our BFO films, besides the crystal structure, tetragonality and interfacial chemical environment, the effective screening from ionic displacements of the bottom oxide electrode and screening charges at the interface and top surface might help to stabilize the polar states at atomic thickness. It has been reported that oxygen vacancy may also be able to induce polarization such as in LAO (LaAlO_3_)/STO system^41^, for which at least 5-u.c. of LAO and very low oxygen growth pressure are required. However, our samples are fabricated under high oxygen pressure and cooled to room temperature under pure oxygen ambient at a slow cooling rate (5 °C min^−1^) to minimize oxygen vacancies. Besides, after electrical poling, the topography of films also appears unaffected as confirmed by AFM, which implies electrochemical phenomena in our experiment is weak^45^. Therefore, oxygen vacancies induced polarization and electrochemical reaction in BFO do not play a major role in our measured ferroelectric behavior.

In summary, the out-of-plane room-temperature ferroelectricity and its switchability down to the two-dimensional limit in tetragonal BiFeO_3_ films were observed. The experimental observations and theoretical calculations suggest that the crystallographic structure, tetragonality, interfacial chemical environment, and the ionic polarization of oxide electrode contribute to reduce or eliminate the critical thickness for ferroelectricity. In addition, a TER ratio of ~370% was achieved in FTJ with 1-u.c. tetragonal BiFeO_3_ tunnel barrier, which shows great promise for high-density data storage. Our findings will open up possibilities for miniaturizing ferroelectric-based devices.

## Methods

### Sample preparation

All ultrathin BFO films were epitaxially grown on atomically smooth (001) STO single-crystal substrates (Crystec GmbH) by laser molecular beam epitaxy (LMBE) technique (KrF laser 248-nm). After deposition, the samples were cooled to room temperature under a pure oxygen ambient at a cooling rate of 5 °C min^−1^ to reduce oxygen vacancy.

### Sample characterization

AFM, PFM, KPFM, and C-AFM measurements were carried out on a commercial scanning probe microscope (Asylum Research MFP-3D) instrument to characterize the morphology, ferroelectricity, distribution of surface electrostatic potential, and nanoscale resistance switching, respectively. Atomically flat atomic force microscopy (AFM) images of STO, SRO, and BFO, with clear terraces separated by ~0.39 nm high steps, respectively, were observed, as shown in supplementary Figure 1. The crystal structure and strain state were characterized by synchrotron XRD using a four-circle diffractometer at the Singapore Synchrotron Light Source (SSLS). The atomically smooth surface and interface were also verified by X-ray reflectivity (XRR) (Supplementary Figure 27). Cross-sectional TEM samples were prepared with a focused ion beam setup (DA300, FEI). The microstructure, thickness, and element distribution of the films were characterized using aberration-corrected STEM at high-angle annular dark-field mode and energy dispersive X-ray mapping on FEI Titan G2 80–200 microscope equipped with a Super-X EDX detector at an emission voltage of 200 kV.

### Others

The schematic crystal structure is produced using VESTA 3^46^. The detailed information of the film growth, characterization, atom position determination, and first-principles calculations are listed in the supplementary section.

### Data availability

The authors declare that all other relevant data supporting the findings of the study are available in this article and in its Supplementary Information file. Access to our raw data can be obtained from the corresponding author on reasonable request.

## Electronic supplementary material

Supplementary Information
