# Supplementary material for: Direct observation of room-temperature out-of-plane ferroelectricity and tunneling electroresistance at the two-dimensional limit
Source: Nat Commun. 2018 Aug 20;9:3319. doi: 10.1038/s41467-018-05662-y (PMC6102252; doi:10.1038/s41467-018-05662-y)
Supplement: Supplementary file 1 — Supplementary Information [file 41467_2018_5662_MOESM1_ESM.docx]

Supplementary Information **“Direct observation of room-temperature out-of-plane ferroelectricity and tunneling electroresistance at the two-dimensional limit”**

Wang *et al*.

**Supplementary Note 1. Summary of Sample Profile and Characterization Method:**

**Sample Profile**

We have grown a number of heterostructure films on cubic SrTiO_3_ (001) substrates as labeled below:

SrTiO_3_ (STO)/SrRuO_3_ (SRO) (~7 nm)/BiFeO_3_ (BFO) (1 u.c.);

STO/SRO (~7 nm)/BFO (2 u.c.), (Figure 1 in main text);

STO/SRO (~7 nm)/BFO (3 u.c.), (Figure 1 in main text);

STO/SRO (~7 nm)/SrO termination/BFO (3 u.c.);

STO/SRO (~7 nm)/BFO (~12 nm);

STO/SRO (~7 nm)/BFO (1 u.c.)/SRO (~7 nm);

STO/SRO (~7 nm)/BFO (2 u.c.)/SRO (~7 nm);

STO/SRO (~7 nm)/BaTiO_3_ (BTO) (2 u.c.);

STO/SRO (~7 nm)/STO (2 u.c.);

STO/SRO (~12 nm).

**Characterization Method**

We have characterized the atomic displacement, ferroelectric switching, surface electrical potential distribution and performance of tunneling electroresistance of ultrathin BFO combining the means of STEM, PFM, KPFM and CAFM.

**STEM section:**

Atomic-scale observation of polarization of STO/SRO (~7 nm)/BFO (2~3 u.c.) in Supplementary Figure 2;

Atomic-scale observation of polarization of STO/SRO (~7 nm)/BFO (1 u.c.) in Supplementary Figure 3;

Atomic-scale observation of polarization of STO/SRO (~7 nm)/BFO (2 u.c.)/ SRO (~7 nm) in Supplementary Figure 4;

Atomic-scale observation of polarization of STO/SRO (~7 nm)/SrO termination/BFO (3 u.c.) in Supplementary Figure 5;

Atomic-scale observation of polarization of STO/SRO (~12 nm) in Supplementary Figure 6;

Atomic-scale observation of polarization of STO/SRO (~7 nm)/BFO (2~3 u.c.) in Supplementary Figure 7;

Strain distribution map of STO/SRO (~7 nm)/BFO (3 u.c.) in Supplementary Figure 10;

**PFM, KPFM and CAFM section:**

Ferroelectricity of STO/SRO (~7 nm)/BFO (1 u.c.) in Supplementary Figure 11;

Ferroelectricity of STO/SRO (~7 nm)/BFO (~12 nm) in Supplementary Figure 12;

Maps of SS-PFM of STO/SRO (~7 nm)/BFO (2~3 u.c.) in Supplementary Figure 13;

The influence of surface screening charge on PFM measurement of STO/SRO (~7 nm)/BFO (2 u.c.) in Supplementary Figure 14;

KPFM of STO/SRO (~7 nm)/BFO (2~3 u.c.) in Supplementary Figure 15;

Time dependent PFM images of STO/SRO (~7 nm)/BFO (2 u.c.) in Supplementary Figure 16;

PFM image written by different DC voltages in Supplementary Figure 17;

SS-PFM measured by different AC voltages in Supplementary Figures 18 and 19;

PFM and CAFM of STO/SRO (~12 nm) in Supplementary Figure 20;

Growth of (2 u.c.) BTO and (2 u.c.) STO films in Supplementary Figure 21;

PFM measurement of BTO (2 u.c.) and STO (2 u.c.) in Supplementary Figures 22 and 23;

PFM measurement of BFO (2 u.c.) with a square Pt top electrode in Supplementary Figure 24;

TER effect of STO/SRO (~7 nm)/BFO (2 u.c.) in Supplementary Figure 25.

**Supplementary Note 2. Sample Preparation details:** The ultrathin films were grown by laser-molecular beam epitaxy (LMBE) technique onto atomically smooth (001) SrTiO_3_ (STO) single-crystal substrates (Crystec GmbH) with a small miscut angle (< 0.5°) buffered by conducting SrRuO_3_ (SRO) as bottom electrodes using KrF (248-nm) laser. STO substrates with a fully TiO_2_ termination were obtained after a buffered hydrofluoric (HF) acid-etch for 30 s and annealing at 975 °C for 3 hours. A laser spot size 4 mm^2^ at a frequency of 3 or 5 Hz was created for ablating the stoichiometric targets (2-inch Bi_0.9_FeO_3_ (BFO) and 1 inch SRO). The substrate was placed at 6.5 cm distance directly above BFO and SRO targets. SRO was deposited as a bottom electrode at substrate temperature 600 °C in a flowing oxygen atmosphere of 100 mTorr by using laser fluence of 1.2 Jcm^-2^. Subsequently, the condition was adjusted to 590 °C and 130 mTorr for BFO by using laser fluence of 1 Jcm^-2^. To prepare SrO terminated SRO electrodes with the same electrical properties and crystal structure, one unit cell SrO is inserted by ablating a SrO target at the same growth conditions with that of SRO. During the growth, in situ high-pressure Reflective high-energy electron diffraction (RHEED) was utilized to monitor the film thickness and verify the growth mechanism. As shown in supplementary Fig. S28, the growth mechanism shows the transition from the layer by layer to step flow. The streaky RHEED pattern in the inset reveals a two-dimension surface of the film. After deposition, the samples were cooled to room temperature under a pure oxygen ambient at the cooling rate of 5 °Cmin^-1^ to reduce oxygen vacancy.

**Supplementary Note 3. Sample Characterizations:** All AFM, PFM, CAFM and KPFM measurements were carried out with commercial scanning probe microscope (Asylum Research MFP-3D) instrument at room temperature by different types of probes. The topography of treated substrates and as-grown films were probed by atomic force microscopy (AFM) mode, while the ferroelectricity of ultra-thin BFO was measured by using piezoelectric force microscopy (PFM) mode with Pt coated tips (AC240TM, spring constant of ∼2 Nm^−1^, Olympus, Japan) to measure the local switching spectrum and out-of-plane PFM image under ambient air and slow-flowing argon gas. The drive frequency, drive amplitude (*V*_AC_) and trigger force are ~270 kHz, 1 V and 80 nN, respectively. After uniform prepoling by an electric field, the distribution of surface electrostatic potential was measured by Kelvin Probe Force Microscopy (KPFM) at a lift height of 30 nm over the film surface. Nanoscale resistance switching has been measured in the conductive atomic force microscopy (CAFM) mode with Pt coated tips (NCHPt). The typical scan rate for all scan modes was 1 Hz. The crystal structures and strain state of our ultra-thin films were characterized by synchrotron X-ray diffraction (XRD) with a four-circle diffractometer (X-ray wavelength of 1.635 Å) at the Singapore Synchrotron Light Source (SSLS). The microstructure, thickness and element distribution of the films were characterized using aberration-corrected scanning transmission electron microscopy (STEM) at high angle annular dark field (HAADF) mode and energy dispersive X-ray (EDX) mapping on FEI Titan G2 80-200 microscope equipped with a Super-X EDX detector at an emission voltage of 200 kV. The cross-sectional samples were prepared by a focused ion beam (FIB) setup (DA300 and Quanta 3D FEG, FEI).

**Supplementary Note 4. Analysis of reciprocal space maps**: To identify crystal and domain structure, 3-dimension reciprocal space mapping (3DRSM) was performed. The reciprocal-space vectors (RSVs) approach^1^ was used to determine Bravais lattice structure and exact lattice parameters of single-crystal epitaxial films by HR-XRD through collecting reciprocal space maps (RSM) around different reflections which are independent to each other. In the determining process, the lattice parameters of substrates are used as references to correct the systematic errors induced by machine. The resolution of this method is 0.001 Å, which is smaller than the error generated by other structure characterization approaches.

**Supplementary Note 5. Determination of cation displacement vector:** BFO is a well-known ionic-displacive ferroelectric material. Theoretical studies reveal that the displacement mainly arises from the displacement of Bi, Fe and O relative to each other^2-4^. The electric dipole moment is from the offset between the center of negative (oxygen) and positive (iron and bismuth) charges. Experimentally, due to difficulty in imaging the light element (O), HAADF-STEM images were widely used to identify displacement vector pattern of cation for the tetragonal structure with negligible oxygen octahedral rotation and shift^3,5-7^. Both A-site and B-site atom display off-center displacement, which induces the elongation of the lattice in the direction of out-of-plane of the film. We define a vector **D**_Fe_, which is the relative displacement between the Fe cation and the mass center of an elongated rectangle formed by its four Bi neighbors, as shown in Supplementary Figure 29. To extract the quantitative displacement vector of the atom, the atom position in STEM image needs to be precisely identified. Firstly, the recorded STEM images are filtered by Fast Fourier Transform (FFT) using only a low-pass annular mask, then the atom positions are accurately determined by fitting them as 2D Gaussian peaks by using Matlab toolbox. The displacement vector of Fe is calculated as a vector from the mass center of nearest neighbor Bi to each Fe. Here, we assume the atom Fe displacement with respect to Bi is the dominant contribution of ferroelectric polarization, which has been proven by some theoretical results^8,9^.

**Supplementary Note 6. The Computational Methods:** The atomic and electronic structure of the system are obtained using density functional theory (DFT) as implemented in Vienna *ab initio* simulation package (VASP)^10,11^. The projected augmented plane wave (PAW) method is used to approximate the electron-ion potential^12^. The exchange and correlation potential is calculated in local density approximation (LDA). In the calculation, we use a kinetic energy cutoff of 340 eV for the plane wave expansion of the PAWs and grid (8x8x1) of k points^13^ for the Brillouin zone integration. The in-plane lattice constant is fixed to that of the substrate SrTiO_3_ while the bulk c/a ratio and the internal atomic coordinates are relaxed until the Hellman-Feynman force on each atom is less than |0.01| eV/Å. The exchange and correlation beyond GGA were taken into account by introducing an onsite Coulomb repulsion with Hubbard U= 3.5 eV^14^ for Fe 3d orbitals in rotationally invariant formalism^15^, as implemented in VASP. For the calculations, we used symmetric slabs of BFO/SRO/BFO and supercells of SRO/BFO/SRO. The calculated A-O and B-O displacement were shown in Supplementary Figure 30.

**
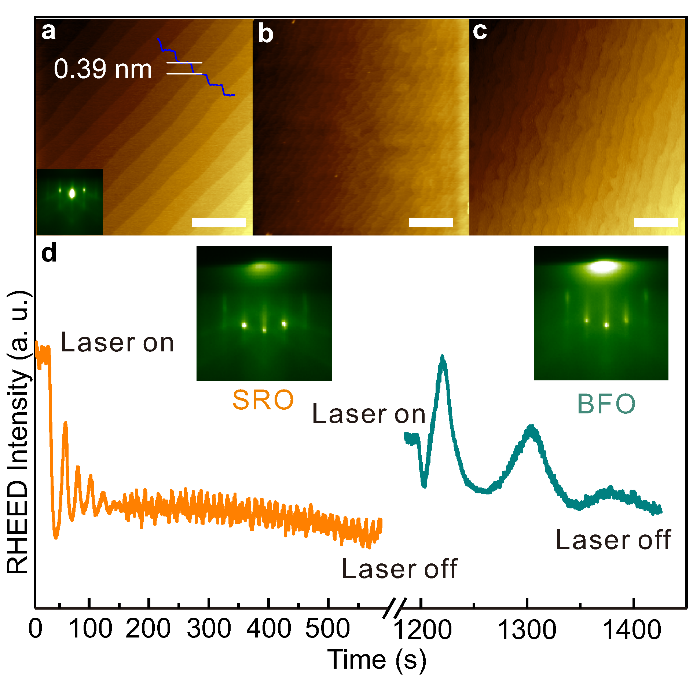
**

**Supplementary Figure 1 | Growth of BFO ultrathin films**. (**a-c**) AFM images of the morphology of (001) TiO_2_-terminated STO substrate (**a**), after SRO deposition on STO (**b**) and after 3-u.c. BFO deposition on SRO/STO (**c**), respectively. Insets of (**a**, **d**) show RHEED patterns of the substrate before and after SRO and BFO deposition respectively. Inset of (**a**), blue line illustrates surface profile of STO substrate. The scale bar is 1 μm. (**d**) RHEED intensity oscillation during the growth of SRO and BFO.

We have grown ultrathin BFO films on top of SRO films on STO substrates. Supplementary Figures 1a-1c show atomically flat AFM image of STO, SRO and BFO, with evident terraces separated by ~0.39 nm high steps. The sharp RHEED patterns shown in the insets of Supplementary Figures 1a and 1d are indicative of excellent crystallinity and a smooth 2D surface. During the growth of SRO and BFO, strong RHEED intensity oscillations monitored by in-situ RHEED (Supplementary Figure 1d) indicates the layer-by-layer growth mechanism.

**
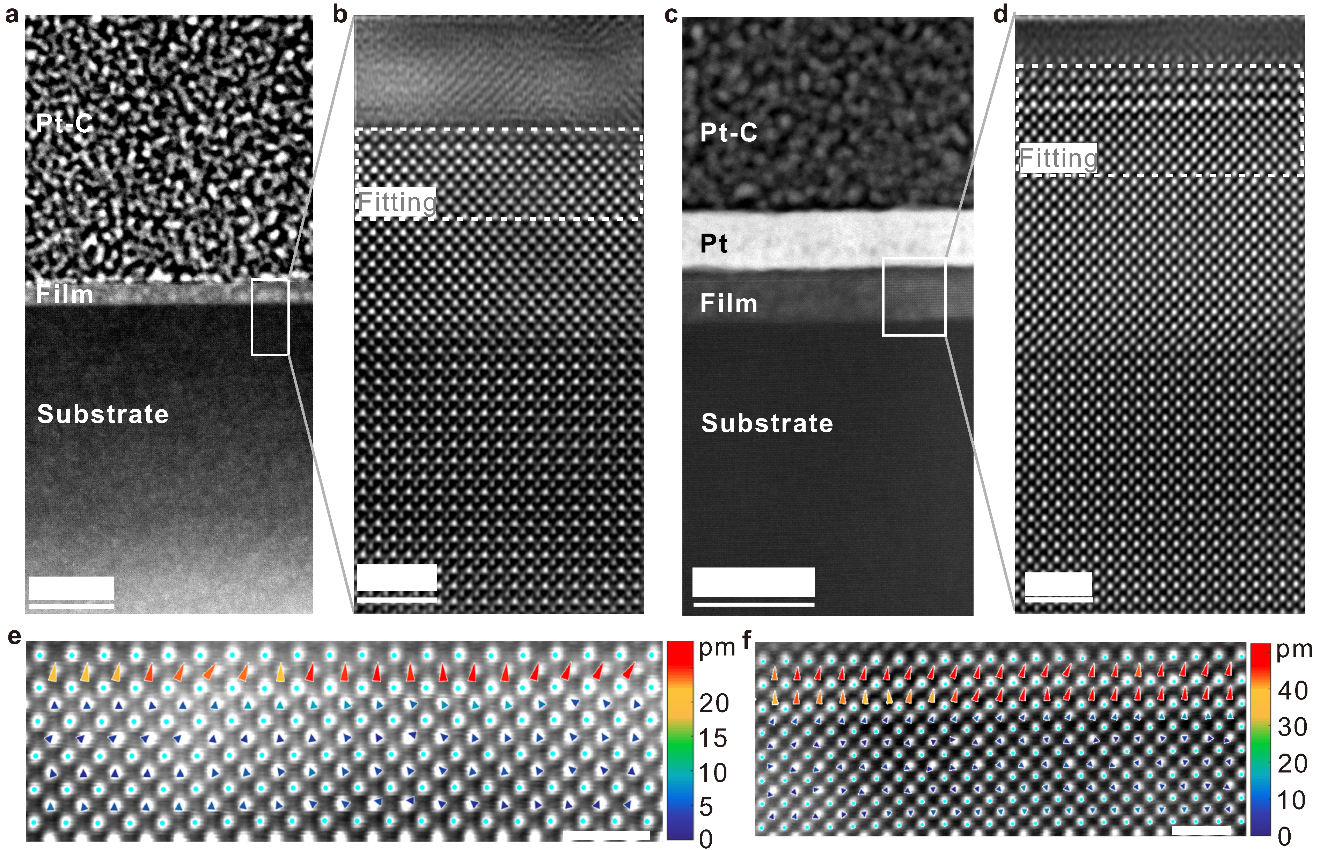
**

**Supplementary Figure 2 | Low-resolution STEM and atomic positon determination**. (**a**, **c**) Full view of 2-u.c. and 3-u.c. BFO film capped by conductive Pt-C layer on SRO buffered STO, respectively. The scale bar is 20 nm. (**b**, **d**) The enlarged images at the region marked by an orange rectangle in (**a**) and (**c**), respectively. The scale bar is 2 nm. (**e**, **f**) Superposition of a magnified image and Fe^3+^ displacement vector maps of 2-u.c. and 3-u.c. BFO respectively. The scale bar is 1 nm.

To show the complete stacking structure of TEM samples, low-resolution STEM images were shown in Supplementary Figure 2. One Pt/Pt-C capping layer was evaporated on BFO/SRO/STO heterostructures for the purpose of fabricating cross-sectional TEM sample by focused ion beam (FIB) process.


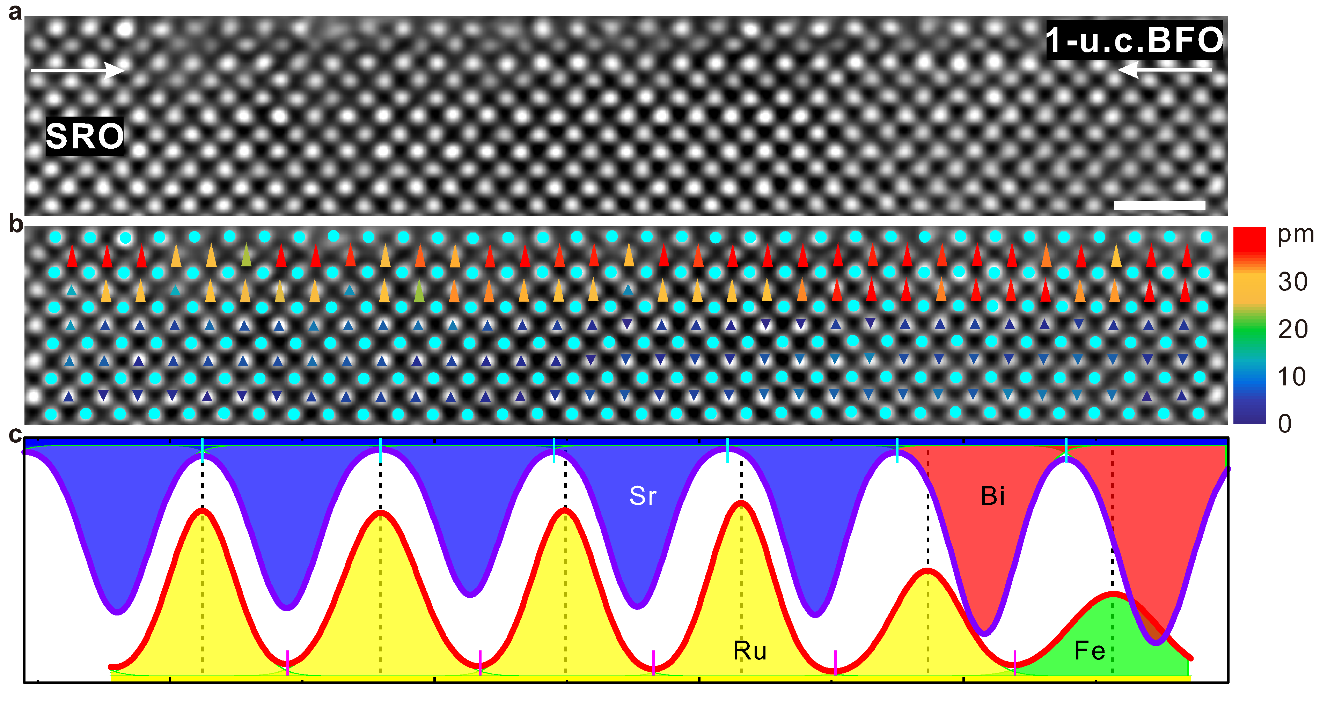


**Supplementary Figure 3 | Atomic-scale observation of polarization in one unit cell BFO on SRO layer.** (**a**) Atomic resolution HAADF-STEM image of 1-u.c. BFO film. The white arrows mark the heterointerface of SRO/BFO. The scale bar is 1 nm. (**b**) Superposition of STEM image and displacement vector map of B-site atoms. The length and direction of the arrows represent the magnitude and direction of the displacement vectors with respect to the scale bar. (**c**) STEM intensity profiles of A-site (purple) and B-site (red) cations across BFO/SRO heterostructure. The cyan short lines represent the center of A-site atoms, while the black dashed lines represent the position of B-site atoms.

The cross-sectional STEM images and atomic displacement vector map of one-unit-cell thick BFO films buffered by SRO layer are shown Supplementary Figure 3. One could observe a noticeable ionic relative displacement in one unit cell BFO films, which is consistent with our PFM results.


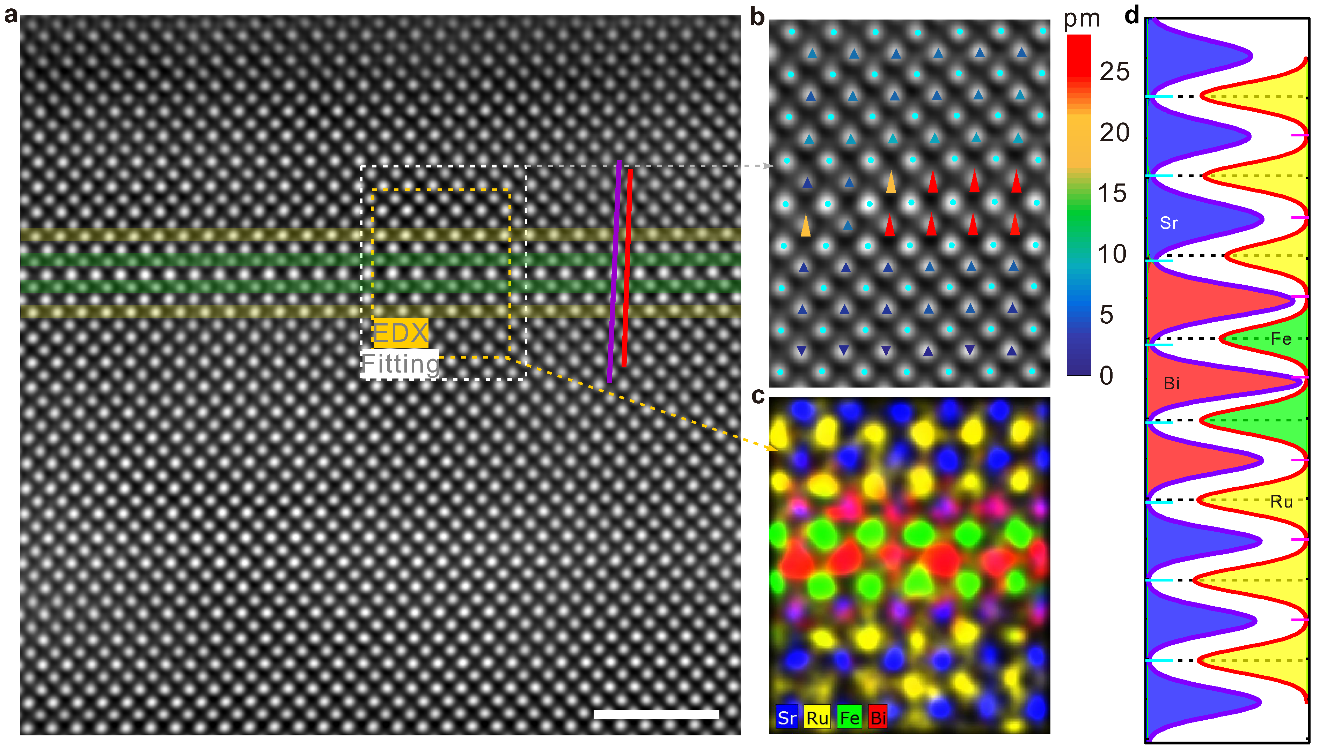


**Supplementary Figure 4 | Atomic-scale observation of polarization in two unit cells BFO sandwiched by two SRO layers.** (**a**) Atomic resolution HAADF-STEM image of SRO/2-u.c. BFO/SRO. The yellow and green lines represent the Ru and Fe atomic layer, respectively. The scale bar is 2 nm. (**b**) Superposition of a magnified image and of B-site atoms from an area marked with a white dashed rectangle in (**a**). The length and direction of the arrows represent the magnitude and direction of the displacement vectors with respect to the scale bar. (**c**) The false-color EDX elemental maps overlays of Bi (red), Fe (green), Sr (blue) and Ru (yellow) from an area marked with an orange dashed rectangle in (**a**). (**d**) The STEM intensity profiles of A-site (purple) and B-site (red) cations across heterointerface of BFO/SRO with SrO and RuO_2_ terminations along the purple and red lines in (**a**). The cyan short lines represent the center of A-site atoms, while the black dashed lines represent the position of B-site atoms.

To verify the spontaneous polarization in ultrathin BFO films, we examine the atomic displacement of BFO in a symmetric environment (SRO/BFO/SRO) using aberration corrected STEM. As shown in Supplementary Figure 4, the BFO films placed in a symmetric environment also have an apparent atomic displacement.


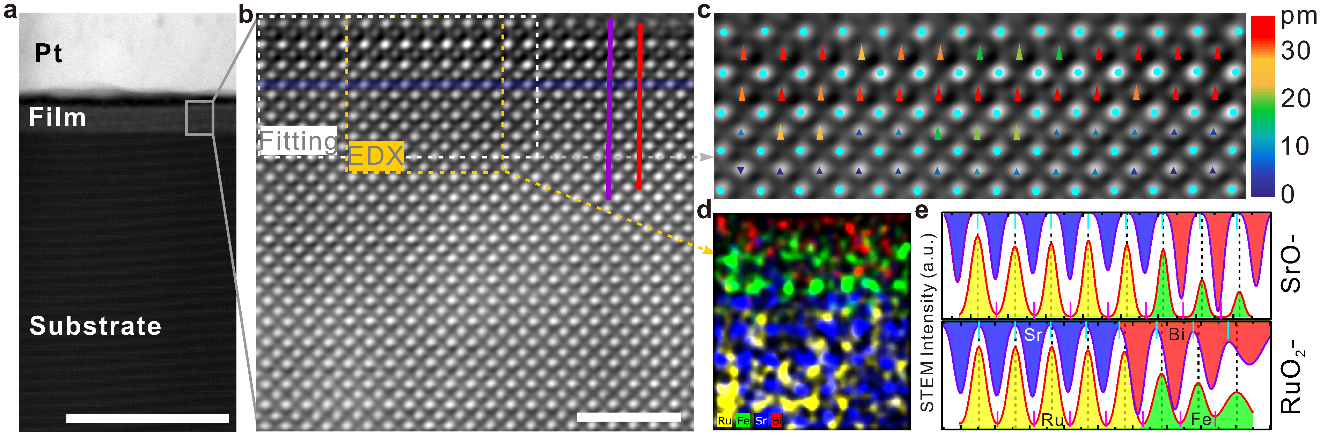


**Supplementary Figure 5 |** **Atomic-scale observation of polarization in three unit cells BFO on SRO layer with SrO termination.** (**a**) A full view of BFO/SRO films capped by protective Pt layer on STO substrate. The scale bar is 50 nm. (**b**) Atomic resolution HAADF-STEM image of SRO area marked by a grey rectangle in (**a**). The scale bar is 2 nm. (**c**) The displacement vectors map from the area marked with a white dashed rectangle in (**b**). (**d**) The false-color EDX elemental maps overlays of Bi (red), Fe (green), Sr (blue) and Ru (yellow) from an area marked with an orange dashed rectangle in (**a**). (**e**) The STEM intensity profiles of A-site (purple) and B-site (red) cations across heterointerface of BFO/SRO with SrO and RuO_2_ terminations along the purple and red line in (**b**).

In order to achieve SrO terminated SRO bottom electrodes with the same conductivity and crystal structure as that of RuO_2_ terminated SRO, one unit cell SrO is inserted by ablating a SrO target between SRO and BFO. Ferroelectric polarization in cross-section along the <100> direction is characterized by the means of STEM, as shown in Supplementary Figure 5. By 2D Gaussian fitting approach, we achieved an accurate value of atomic displacement, as shown in Supplementary Figure 5c. The EDX mapping in Supplementary Figure 5d verifies that the atomic stacking at the SRO/BFO interface is -SrO-FeO_2_-. An apparent atomic displacement could be also observed in SrO terminated SRO bottom layers. To perform a visual comparison, the STEM intensity profile across two SRO/BFO interfaces are shown in Supplementary Figure 5e. In comparison, the Fe ion displacement of BFO on SrO termination is about 25 pm, while the value of BFO on RuO_2_ termination is up to 35 pm. For the former, 2-3 unit cells of SRO are polarized to screen a depolarizing field, while only 1-2 unit cells of SRO are polarized for the latter.


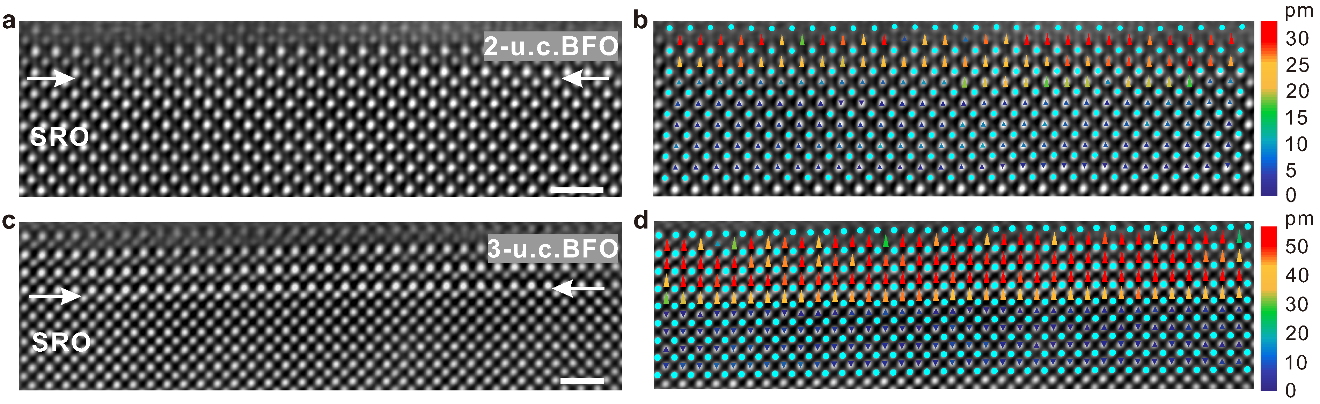


**Supplementary Figure 6 |** **Atomic-scale observation of polarization of BFO on SRO buffered (001) STO substrate.** (**a, c**) Atomic resolution HAADF-STEM images of 2-u.c. and 3-u.c. BFO film in other samples. (**b, d**) The displacement vectors map of (**a, c**). The scale bar is 1 nm.

As well known, TEM measurements are much localized in order to accurately determine the displacement of atoms with sub-angstrom resolution. To ensure the experimental results that we observe are consistent across our sample, we can only try our best to repeat the measurements on more samples and different regions across our sample. More than 20 areas from different regions have been checked carefully and all BFO films show similar atomic displacement in all those regions. STEM images and their displacement vector mapping from other regions are shown in Supplementary Figure 6 for comparison purposes.


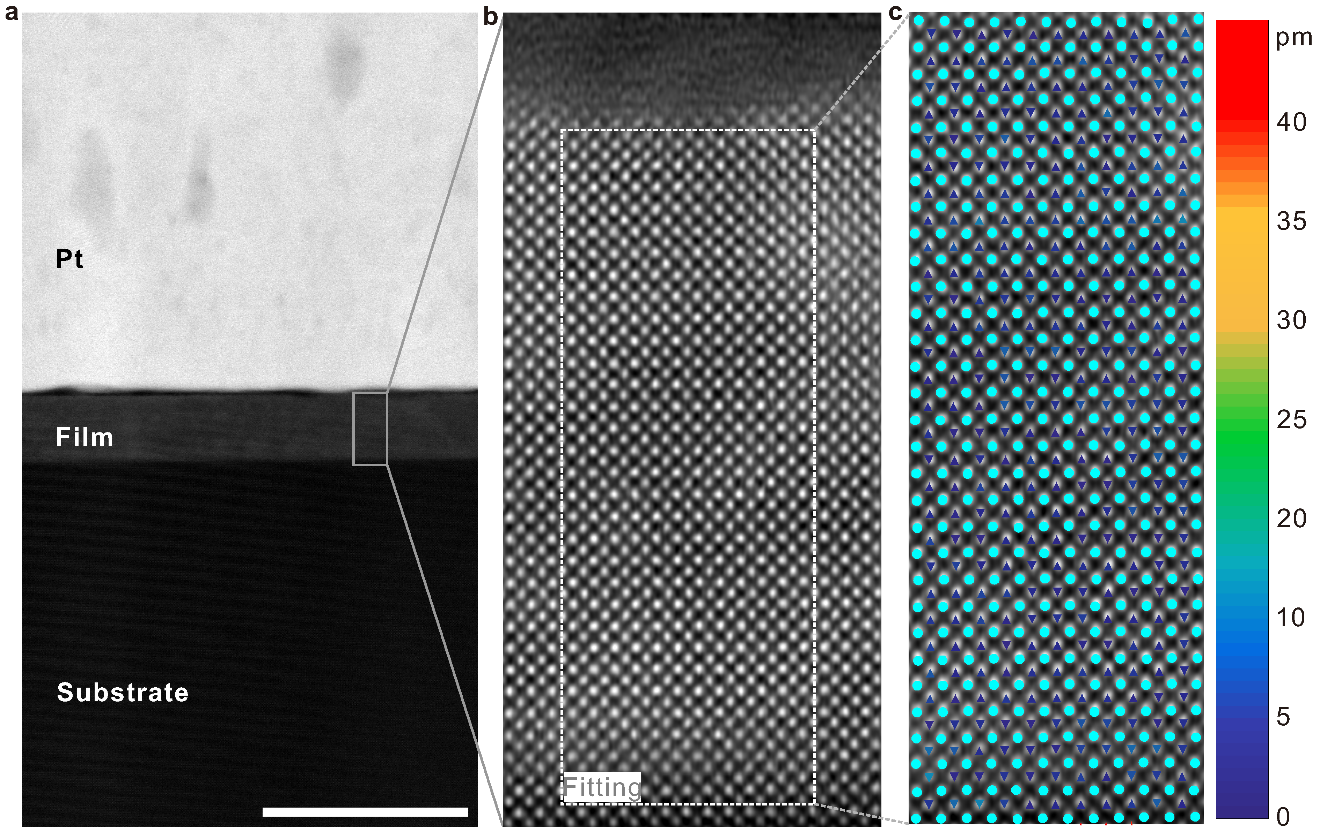


**Supplementary Figure 7 | HAADF-STEM image of SRO layer without a BFO capping layer on (001) STO.** (**a**) A full view of SRO film capped by protective Pt layer on STO substrate. The scale bar is 50 nm. (**b**) Atomic resolution HAADF-STEM image of SRO area marked by a grey rectangle in (**a**). (**c**) The displacement vectors map from the area marked with a white dashed rectangle in (**b**).

We have checked the cross-section STEM image of SRO layer without a BFO capping layer along the <100> direction. As shown in Supplementary Figure 7, the tetragonality (*c*/*a*) of SRO film is measured to be 1.02~1.03, whereas, atomic displacement in SRO is less than 5 pm according to the measurement in Supplementary Figure 7c, which agrees with the centrosymmetric structure of bulk SRO. Thus there’s no intrinsic ionic displacement in the SRO layer without BFO film growth upon it. The off-center displacement of Ru atoms near the SRO/BFO surface is caused by the polarization of BFO films.

**
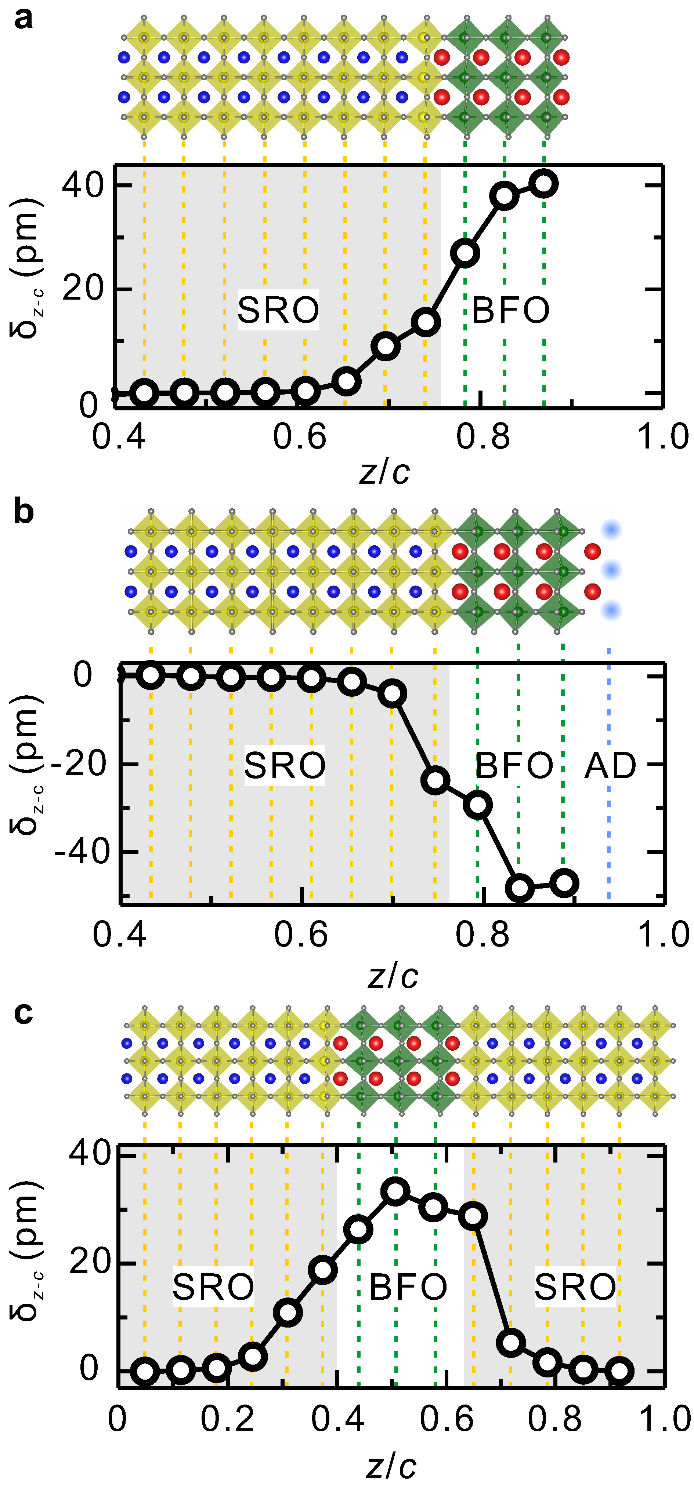
**

**Supplementary Figure 8 | Structure and off-center displacement of 3-unit-cell tetragonal BFO in asymmetric and symmetric SRO environment.** (**a**) Vacuum/BFO/SRO heterostructure. (**b**) Vacuum/adsorbed oxygen (Ad)/BFO/SRO heterostructure. (**c**) SRO/BFO/SRO heterostructure. Top panels: Simulation cells. Bottom panels: Off-center displacement along the growth direction of the lattice.

To further investigate the influence of asymmetric, symmetric environment and surface screening charge on ferroelectricity of ultrathin BFO films, we performed additional first principles calculations. Supplementary Figure 8 shows the calculated off-center displacement for different environments of the 3-u.c. BFO layer. In all the calculations, the BFO is assumed tetragonal and stoichiometric and the BFO/SRO termination is assumed to be BiO/RuO_2_. The appearance of polarization in tetragonal BFO is seen from the relative displacement of Bi and Fe ions similar to that observed in bulk rhombohedral BFO. For the vacuum/BFO/SRO heterostructure (Supplementary Figure 8a), the presence of positively charged top (BiO)^1+^ monolayer supports the spontaneous polarization. The surface polarization charge to a large extent is compensated by the ionic charge of the (BiO)^1+^ terminated surface. The polarization charge at the BFO/SRO interface is screened by electron accumulation in metallic SRO, as well as polar ionic displacement propagating into the bulk SRO layer. The polarization of BFO can be switched if the surface ionic charge on the BFO surface is annulled or reversed, which may occur due to absorption or desorption on the BFO surface. To illustrate this possibility, we added an oxygen atom atop the BFO/SRO heterostructure and calculated the resulting off-center displacement. As shown in Supplementary Figure 8b, the relative displacement changes sign indicating polarization reversal from that of the pristine heterostructure Supplementary Figure 8a). Note that we used oxygen ions for illustration purpose only and using any other electronegative elements would have the same effect. Screening by surface charges is consistent with our Kelvin probe force microscopy measurements. In presence of the top SRO layer (Supplementary Figure 8c), the relative displacement in the BFO layer has similar magnitude as that of the pristine heterostructure (Supplementary Figure 8a). In this case, however, the top SRO layer provides additional screening, as seen in Supplementary Figure 8c from the polar displacement in SRO close to the top SRO/BFO interface.


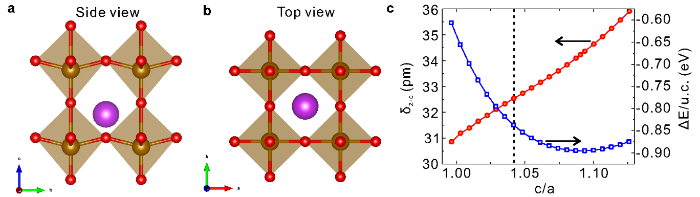


**Supplementary Figure 9 | A full view of structure and energy gain by ferroelectric BFO with respect to centrosymmetric structure by off-centering.** (**a**) Side and top view of BFO unit cell showing displacement of Bi and Fe atoms. (**b**) Off-center displacement as function of c/a (in red) and energy difference between ferroelectric phase and paraelectric phase (in black). The dashed black line locates the observed c/a in experiment.

In Supplementary Figure 9a, we plot the relaxed structure of tetragonal BFO, which clearly shows a relative displacement along (001) direction. Adjacent Supplementary Figure 9b, shows energy gain (Δ*E*) with respect to the centrosymmetric structure by such off-centering (*δ*_z-c_). The figure clearly shows the tetragonal BFO with c/a of 1.05 gains significant energy ~0.54 eV per unit cell with respect to the paraelectric phase due to off-centering and volume increase.


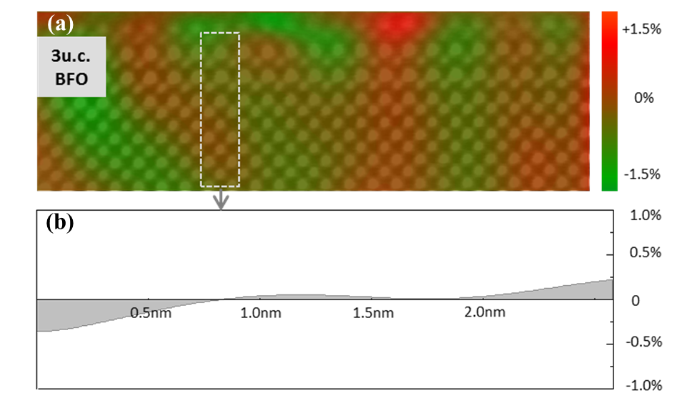


**Supplementary Figure 10 |** **The strain state of BFO on SRO buffered (001) STO**. (**a**) Superposition of STEM image of 3-u.c. BFO and strain distribution map of *ε*_xx_. (**b**) Intensity profile of strain in the white dashed rectangle area in (**a**). [100] is defined as *x*-axis. The color scale bar shows the degree of strain.

Indeed, there are a mass of reported works about the influence of stain on ferroelectricity in thick or ultrathin films. However, strain is not the primary factor to the ferroelectricity observed in two-dimensional BFO films. In the present work, the lattice mismatch between STO and BFO is only 1.4%. Due to the same tetragonal structure of BFO and SRO further reduces their mismatch, the strain at the interface is only 0.88%, as shown in Supplementary Figure 10.


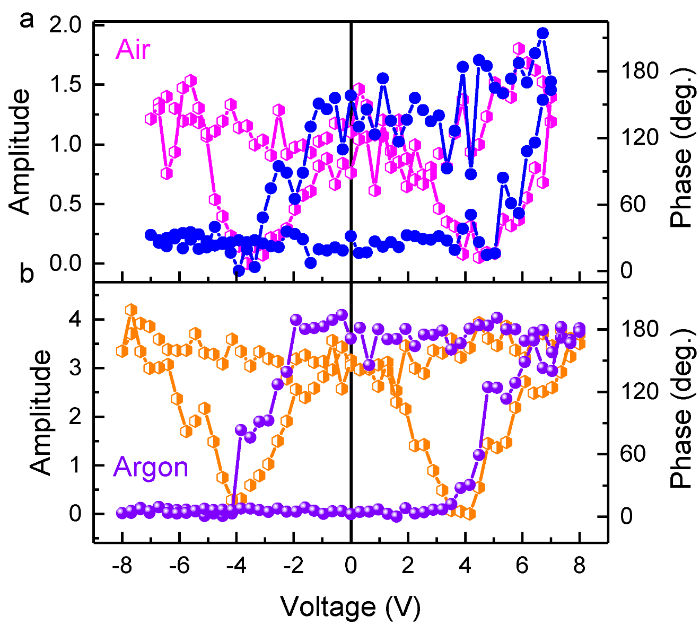


**Supplementary Figure 11 | Ferroelectricity of one unit cell BFO buffered by SRO**. (**a**) Out-of-plane SS-PFM amplitude (half-filled purple hexagon) and phase (filled blue circle) loop measured in air. (**b**) Out-of-plane SS-PFM amplitude (half-filled orange hexagon) and phase (filled violet ball) loop measured in argon.

The OP ferroelectricity of 1-u.c. BFO was also examined by SS-PFM in air (Supplementary Figure 11a) and argon (Supplementary Figure 11b) atmospheres. When the thickness is down to 1-u.c., ferroelectricity becomes weak. The max amplitude signal and the phase difference of ferroelectric (FE) polarization in air is only 30 and 150º, respectively. The typical butterfly-like amplitude and hysteresis phase (the average coercive voltage ~4.6 V) loops reveal stable and switchable ferroelectricity existing in 1-u.c. BFO. Comparing with the results in air, the FE properties in argon are stronger, with an amplitude of 70 and a phase difference of 180º. The average coercive voltage is about 4 V, which is close to that of 3-u.c. BFO. Generally, the content of oxygen vacancy is much lower in atomic-thick oxide film than that of a thick film. This is the reason why the switchable electromechanical response disappears when the LAO thickness is below 5-unit cells^16^. In contrary, the ferroelectricity of BTO, PbTiO_3_ (PTO) and BFO can persist down to 3 unit cells. In addition, the spontaneous piezoresponse signal of hysteresis loops that are originated from non-ferroelectric mechanism decreases with increasing thickness, due to the increased electric field spreading in the LAO layers with larger thickness^16^. However, one knows that the spontaneous polarization signal in ferroelectric film increases with thickness, due to increased permanent electric dipole. Therefore, though oxygen vacancy inevitably exists in oxide films, the motion of Fe cation is still the primary origin of ferroelectricity in the BFO/SRO.

**
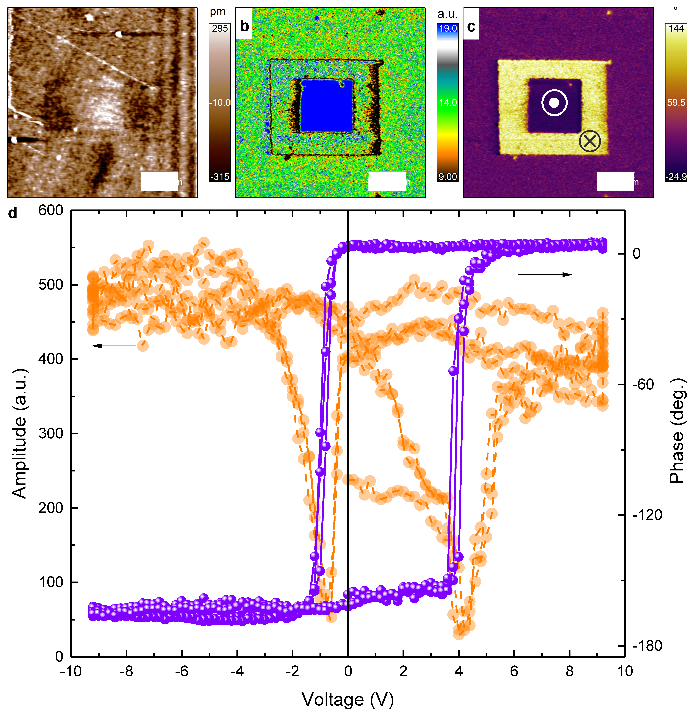
**

**Supplementary Figure 12 | Out-of-plane PFM and SS-PFM results of 12nm BFO buffered conductive SRO measured in air**. (**a**) Topography. (**b**, **c**) PFM amplitude and phase after electrical poling 2*2 μm^2^ with +5 V and 1*1 μm^2^ with -5 V on the bare BFO film, respectively. The scale bar is 800 nm. (**d**) Amplitude (brown filled circle) and phase (purple ball) of SS-PFM between -9 V and 9 V.

Supplementary Figure 12 shows the out-of-plane (OP) ferroelectricity of thick BFO film (about 12 nm) in air. The topography (Supplementary Figure 12a) shows an atomically smooth surface with a roughness of 170 pm, which is consistent with the two-dimensional growth of BFO film. The OP-PFM amplitude and phase were shown in Supplementary Figures 12b and 12c, respectively. Clear amplitude difference and distinct contrast of ~180º between negative/positive poling regions and non-poling region were observed. Combining with the SS-PFM results (Supplementary Figure 12d) which display a butterfly-like amplitude curve (max amplitude of 500) and hysteresis phase loop (coercive voltage of 2.5 V, the built-in field of 1.8 V), we confirmed the great FE nature in the 12 nm BFO film.

**
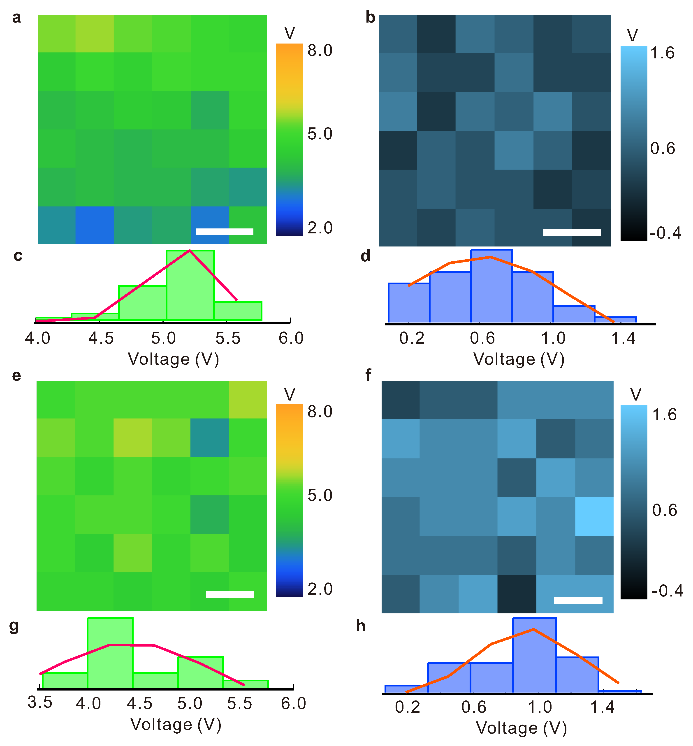
**

**Supplementary Figure 13 | Maps of SS-PFM at 6*6 μm^2^ and 20*20 μm^2^ areas**. (**a**) Coercivity image and (**c**) its histogram and (**b**) Build-in voltage image and (**d**) its histogram of 2-u.c. BFO measured in argon, respectively. The scale bar is 2 μm in (**a**) and (**b**). (**e**) Coercivity image and (**g**) its histogram and (**f**) Build-in voltage image and (**h**) its histogram of 3-u.c. BFO measured in argon, respectively. The red lines show the Gaussian fitting for the data. The scale bar is 5 μm in (**e**) and (**f**).

To confirm the homogeneity of polarization switching in larger scale, we recorded the maps of SS-PFM shown in Supplementary Figure 13 at 6*6 and 20*20 μm^2^ areas on 2-u.c. and 3-u.c. BFO under argon atmosphere, respectively. Through Gaussian fitting for the histograms of coercive voltage and built-in voltage, we achieved the average coercive voltage of 5.2 V and average build-in voltage of 0.7 V for 2-u.c. BFO, while the average coercive voltage of 4.5 V and average build-in voltage of 0.9 V for 3-u.c. BFO.


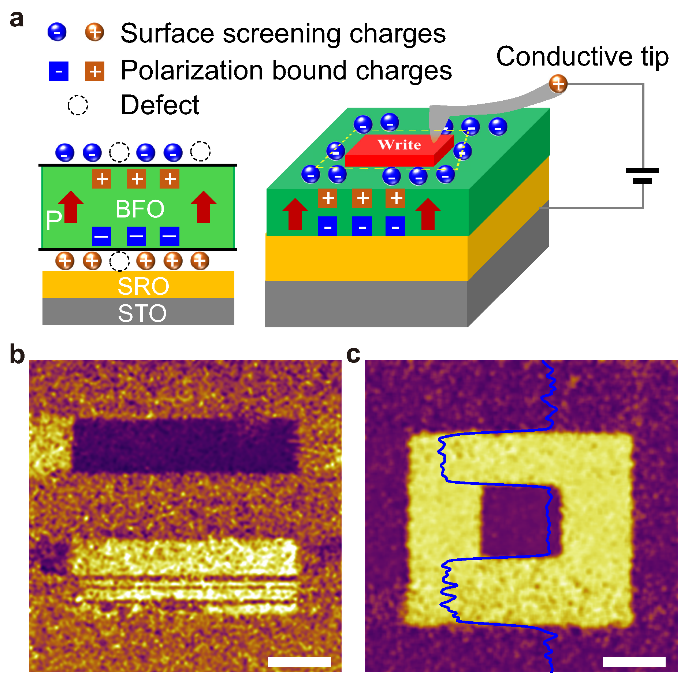


**Supplementary Figure 14 |** **The influence of surface screening charge on PFM measurement of ultrathin ferroelectric film.** (**a**) Schematic illustration of surface screening charge and PFM measurement. (**b**) PFM measurement using conventional sequence. (**c**) PFM measurement using a new sequence. The blue line represents PFM phase profile. The scale bar is 1 μm.

With decreasing ferroelectric film thickness, especially for several unit-cells-thick film, surface screening charges have a non-negligible contribution to PFM measurements. Usually, one first applies a positive and negative DC voltage using a conductive tip on bare ferroelectric film to polarize it, then a small ac voltage is used to image the PFM domains. In both the processes, both surface screening charges and polarization bound charges, as shown in Supplementary Figure 14a, contribute to the PFM signals, which could produce a weak phase contrast, as shown in Supplementary Figure 14b and Fig. 3 in the main text. This phenomenon is typical in ultrathin ferroelectric films, because of the surface screening charges, such as PFM images of 1-nm BTO^17^, 3.2-nm BTO^18^, 1.6-nm PTO^19^, 2-nm La doped BiMnO_3_^20^, 3-nm PbZr_0.52_Ti_0.48_O_3_ (PZT) ^21^ and 3-nm Sm doped BFO^22^. To reduce the effect of such surface screening charges, we utilize a new sequence to record PFM images. First, we scan a large area marked by a white dashed square in Supplementary Figure 14a on the film surface three times by contact mode using conductive tip without any voltage bias. This step could be used to remove surface screening charges through a friction process^23^. Second, a positive DC voltage is applied to a 3*3 μm^2^ region and a negative DC voltage is applied on a 1*1 μm^2^ region. Third, similar to step 1, another three times of contact mode scan without any tip bias are carried out again after application of the DC voltages. Finally, PFM signals are recorded by applying a small ac voltage. The PFM phase image in Supplementary Figure 14c recorded using this sequence shows a better phase contrast as compared to before.

**
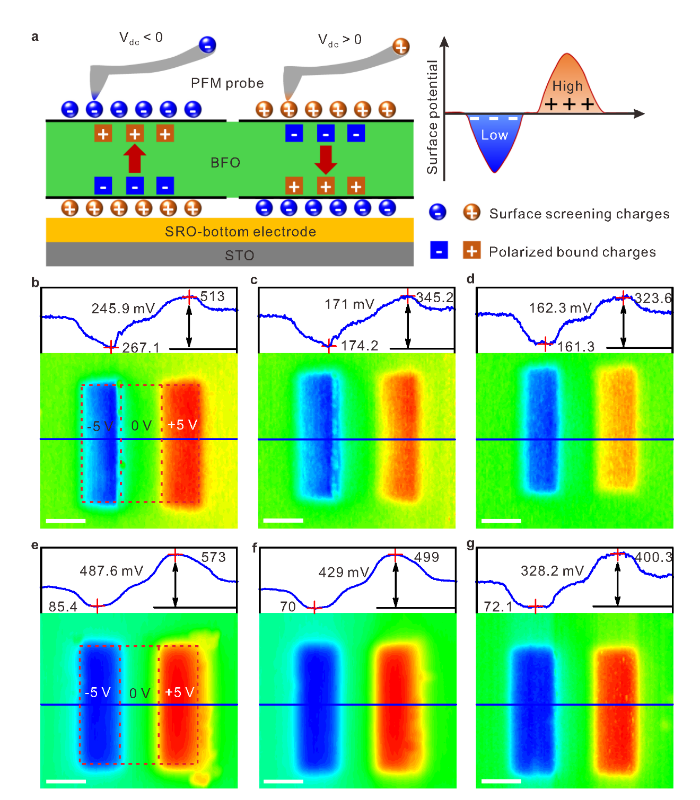
**

**Supplementary Figure 15 | Surface potential distribution measured by KPFM**. (**a**) The schematic illustration of KPFM measurement and screening mechanism. (**b-d**) The potential profiles along the blue lines and surface potential maps of 2-u.c. BFO after poling +5 V, 0 V and -5 V voltage rectangle measured in argon (**b**), in air (**c**) and after three time tip-grounded scans in air (**d**), respectively. (**e-g**) The line profiles at blue lines and surface potential maps of 3-u.c. BFO poled by +5 V, 0 V and -5 V voltage rectangle measured in argon (**e**), in air (**f**) and after three time tip-grounded scans in air (**g**), respectively. The red dashed lines mark the poled areas. The scale bar is 1 μm.

To understand the charge distribution and screening mechanism of ultrathin BFO films, KPFM characterization was carried out in air and argon atmosphere immediately after electrical poling. Supplementary Figure 15a illustrates the schematic of KPFM setup and screening mechanism. To minimize the free energy of FE system, bound charges from FE polarization will be compensated by intrinsic/extrinsic screening charges. Therefore, both the screening charges at the interface of FE/electrode and the bound charges contribute to the KPFM signals. Compared with the electric potential of the unpoled area, the negatively (positively) poled area exhibits a lower (higher) surface potential because the bound charges are overcompensated by injected charges through the conductive probe. As shown in Supplementary Figures 15b-15g, KPFM amplitude in the poled region is distinct from the magnitude in the unpoled region. The difference in surface potential at the negatively/positively poled area under argon gas atmosphere (Supplementary Figure 15b) is about 246 mV which is larger than 171 mV measured in air atmosphere (Supplementary Figure 15c). KPFM image after three contact mode scans by a grounded tip in air (Supplementary Figure 15d) illustrates a slightly smaller potential difference, because screening charges on the surface are removed by contacting the film surface^23^. Similarly, the KPFM results of 3-u.c. BFO are shown in Supplementary Figures 15e-15g. Undoubtedly, the surface potential difference of 3-u.c. BFO is larger than that of 2-u.c. BFO both in argon or air atmosphere because of more significant FE polarization of the former one. The surface potential of ultrathin BFO film was dramatically affected by atmosphere, which was attributed to the change of screening charge due to the humidity and oxygen content of the atmosphere. In addition, it has been demonstrated that the electrical field not only switches FE polarization but also induces charge injection^23,24^. The KPFM results reveal that the depolarization field arising from FE polarization is fully compensated by injected charges at the surface, which assists to switch FE polarization in ultrathin BFO.


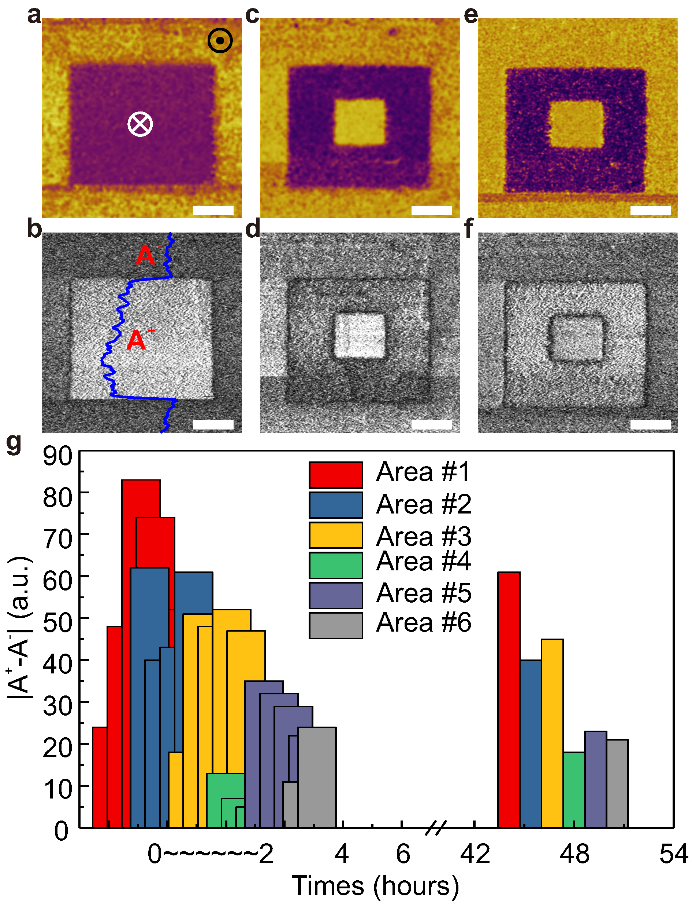


**Supplementary Figure 16 | Time-dependent out-of-plane piezoresponse force microcopy on two unit cells BFO film.** (**a**, **b**) PFM phase and amplitude images after applying +5 V voltage in 3*3 μm^2^, respectively. The blue line in (**b**) represents the line profile from un-poled region to the poled region. A^-^ and A^+^ represent the amplitude value of un-poled and poled region, respectively. (**c**, **d**) PFM phase and amplitude images after applying +5 V voltage in 3*3 μm^2^ and -5 V voltage in 1*1 μm^2^, respectively. (**e**, **f**) PFM phase and amplitude images after 48 hours, respectively. The scale bar is 1 μm. (**g**) The absolute PFM amplitude difference (|A^+^- A^-^|) over time at six random areas.

In order to examine the stability of the oppositely polarized state in ultrathin BFO films, we have recorded PFM signals with time. As shown in Supplementary Figures 16a-16f, the polarized ferroelectric polarization could be switched back by an opposite voltage and the oppositely polarized state could persist over 48 hours. Supplementary Figure 16g shows the PFM amplitude difference decreases by few (10%) percent in the first two hours after electrical poling, and then almost remains constant until 48 hours. Non-ferroelectric signals will gradually vanish with time. Note that the relative large lifetime of the ferroelectric-like signal from non-ferroelectric HfO_2_ is only 1000s (<0.5 hour)^25^.


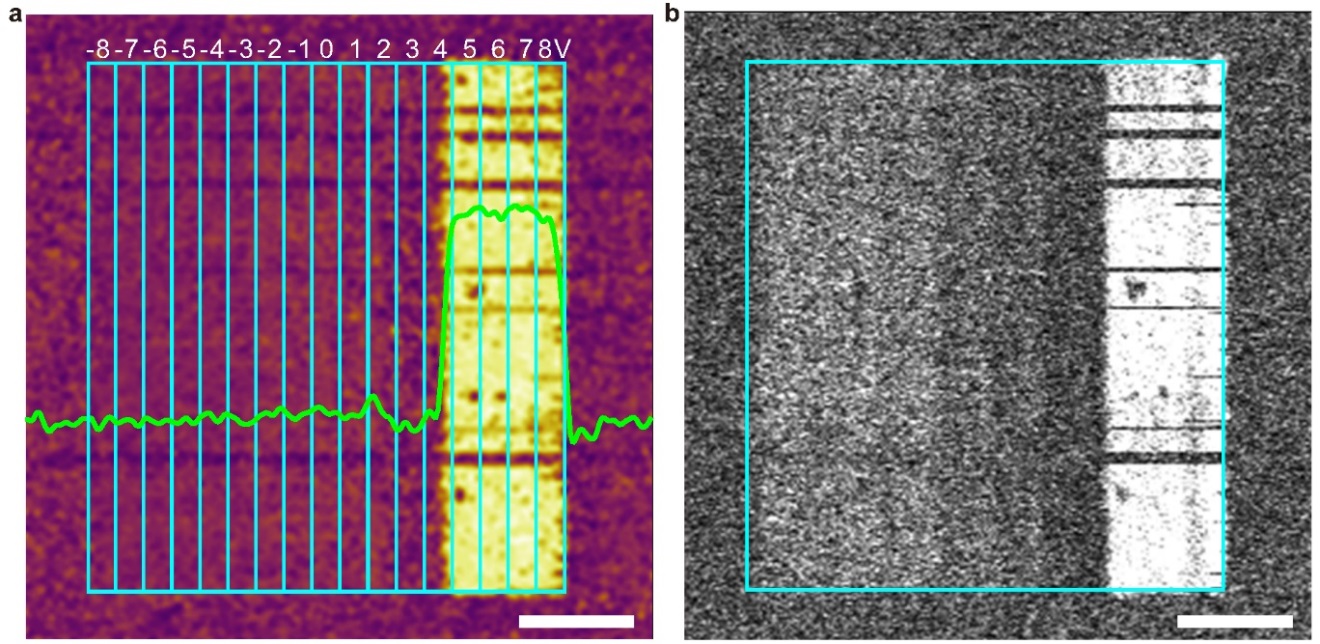


**Supplementary Figure 17 |** **Piezoresponse force microscopy images of two unit cells BFO on SRO buffered (001) STO after poling by different DC voltages.** (**a**) PFM phase image after poling from -8 V to 8V. The cyan rectangle marks the poling area. The green line represents the phase profile. (**b**) PFM amplitude image. The scale bar is 2 μm.


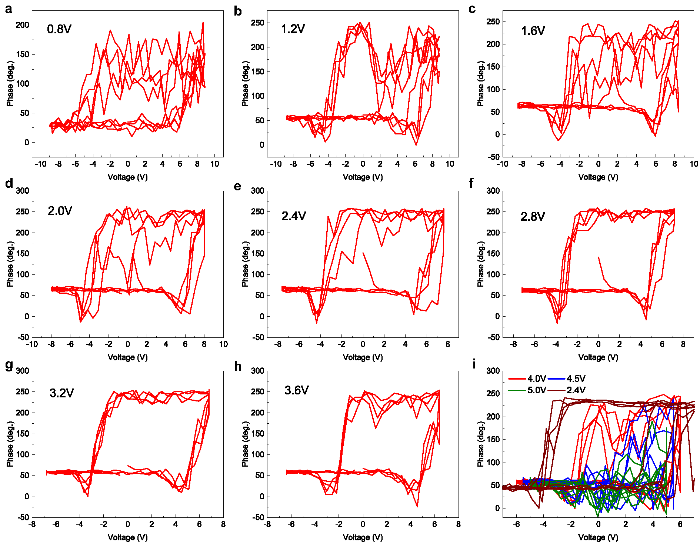


**Supplementary Figure 18 | Local hysteresis curves of two unit cells BFO on SRO buffered (001) STO measured by different AC voltages.** (**a-h**) SS-PFM phase loops under 0.8, 1.2, 1.6, 2.0, 2.4, 2.8, 3.2 and 3.6 *V*_AC_ voltage, respectively. The phase loops at the range of 4.0~5.0 and 2.4 after 5.0 *V*_AC_ voltage measurement are shown in (**i**).


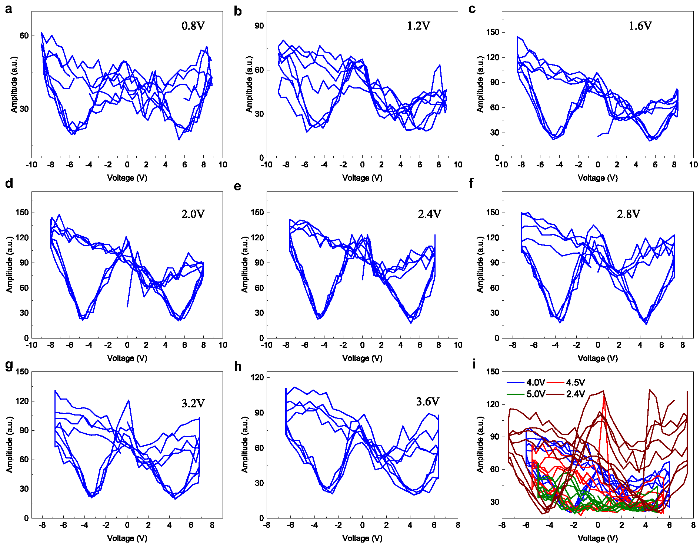


**Supplementary Figure 19 | Local hysteresis curves of two unit cells BFO on SRO buffered (001) STO measured by different AC voltages.** (**a-h**) SS-PFM amplitude curves under 0.8, 1.2, 1.6, 2.0, 2.4, 2.8, 3.2 and 3.6 *V*_AC_ voltage, respectively. The amplitude curves at the range of 4.0~5.0 and 2.4 after 5.0 *V*_AC_ voltage measurement are shown in (**i**).

As well known, many non-ferroelectric mechanisms also contribute to PFM signals, such as surface charges and interface trapped charges and field induced-ion redistribution^16,26,27^. To rule out these non-ferroelectric mechanisms, various PFM experiments are proposed, such as PFM under different DC and AC voltages, conductive grounded-tip scan and contact Kelvin probe force microscopy^26,28,29^. In the present experiment, we also employ these usual approaches to verify the stability and switchability of the polar state. The PFM images recorded after different DC voltage poling (Supplementary Figure 17) and hysteresis curves measured at different AC voltages (Supplementary Figures 18 and 19) reveal that the PFM signals are mainly from ferroelectric polarization switching, which are the signature features of ferroelectric materials.

**
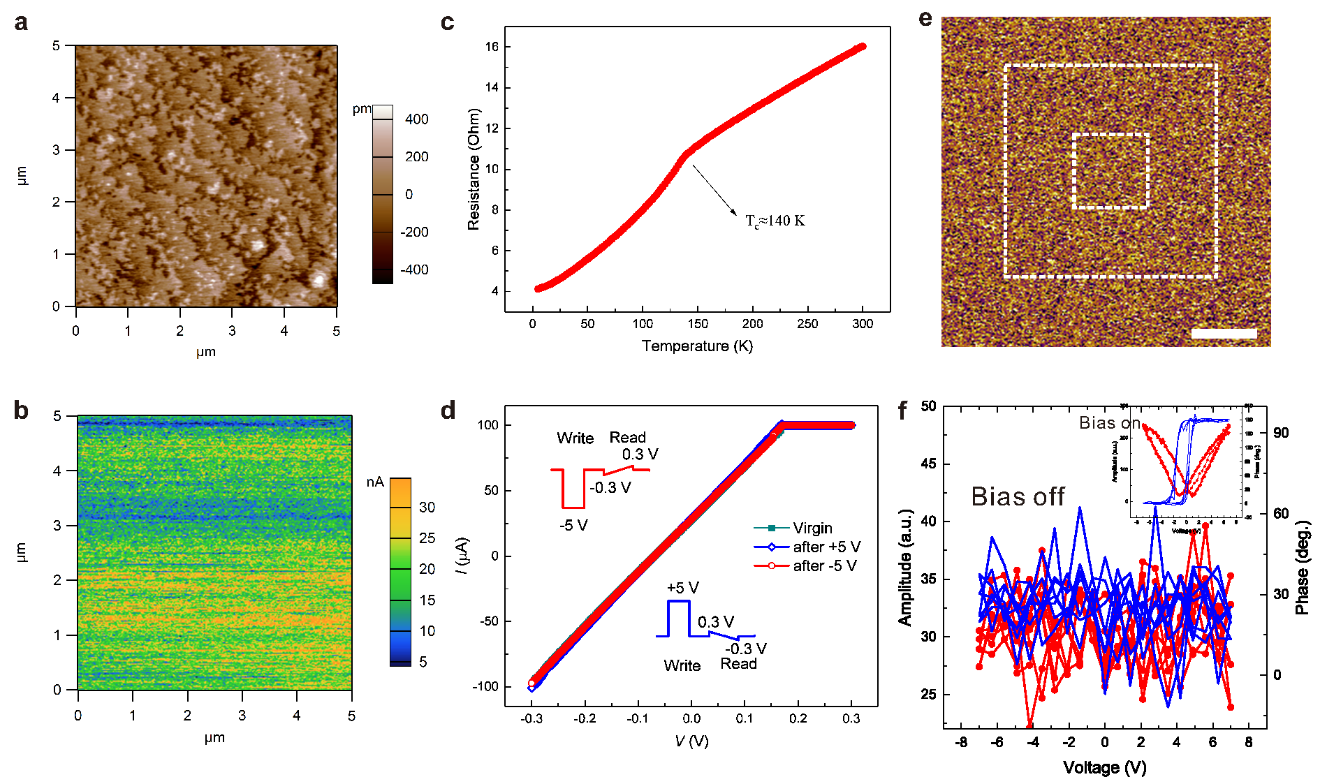
**

**Supplementary Figure 20 | Electric properties and ferroelectricity of SRO electrode**. (**a**) The topography of SRO. (**b**) Current mapping. (**c**) The resistance-temperature curve of SRO between 10 and 300 K. (**d**) *I*-*V* curves on virgin state and after the same write and read voltage sequence with *I*-*V* measurement of BFO/SRO/STO. (**e**) Out-of-plane PFM phase image after writing +/-5 V voltage on the white dashed rectangle area. The scale bar is 1 μm. (**f**) Local out-of-plane SS-PFM amplitude (blue line) and phase (red filled circles) in 4 cycles. The inset of (**f**) shows the bias-on PFM hysteresis curves.

Supplementary Figure 20a shows the smooth topography of 7 nm SRO on STO and clearly atomic step terrace. The root mean square (RMS) surface roughness is about 139 pm. Current mapping of the as-grown SRO shown in Supplementary Figure 20b was acquired by conductive atomic force microscopy (CAFM). The current distribution is very homogeneous except the top edge where the probe is first engaged to the sample surface. Resistance-temperature (R-T) curve shown in Supplementary Figure 20c was measured by four-point probe method on PPMS between 10 and 300 K. The measured R-T curve is consistent with the typical temperature dependence of resistance in SRO^30^. Current-voltage (*I*-*V*) curves (Supplementary Figure 20d) of SRO electrode measured by CAFM after different electrical poling are also linear, revealing a typical perfect ohmic behavior. As expected, for conductive SRO, there is no any ferroelectric signals in PFM images and PFM hysteresis curves as well, as shown Supplementary Figures 20e and 20f.


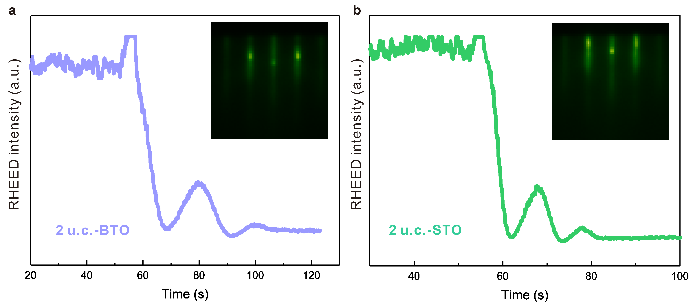


**Supplementary Figure 21 | Growth of two unit cells BTO and STO on SRO buffered (001) STO.** (**a**) RHEED intensity oscillation during the growth of BTO. The inset of (**a**) shows the RHEED pattern after BTO deposition. (**b**) RHEED intensity oscillation during the growth of STO. The inset of (**b**) shows the RHEED pattern after STO deposition.


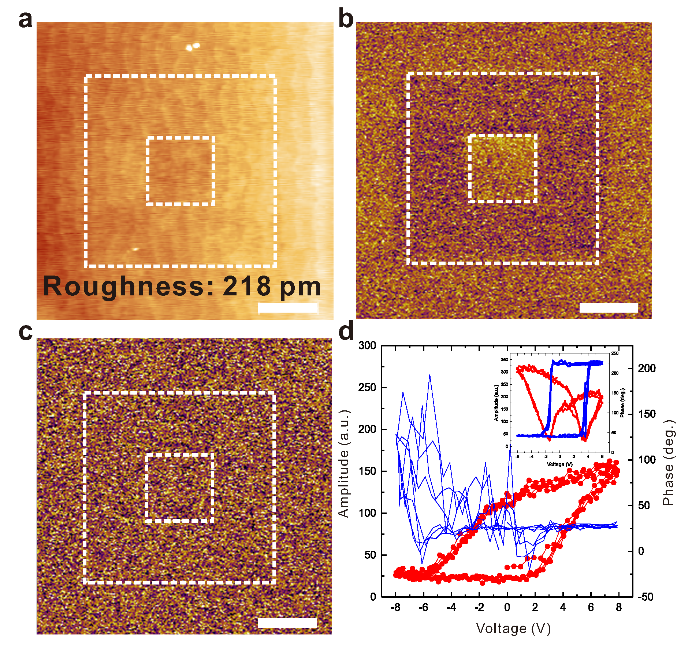


**Supplementary Figure 22 | Piezoresponse force microscopy characterization of two unit cells BTO on SRO buffered (001) STO.** (**a**) AFM image of BTO. (**b**) Out-of-plane PFM phase image after writing +/-5 V voltage on the white dashed rectangle area. (**c**) Out-of-plane PFM phase image after three times conductive tip-grounded scans. (**d**) Local out-of-plane SS-PFM amplitude (blue line) and phase (red filled circles) in 4 cycles. The inset of (**d**) shows the local PFM hysteresis curves of five unit cells BTO. The scale bar is 1 μm.


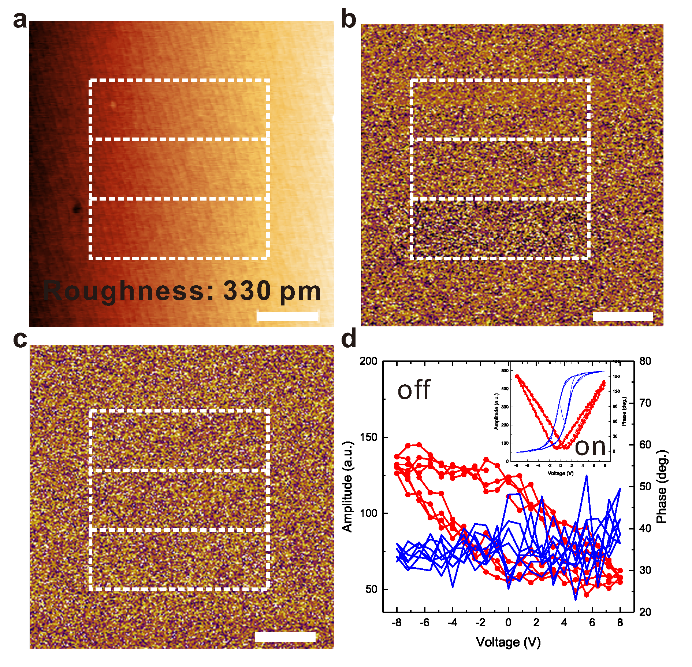


**Supplementary Figure 23 | Piezoresponse force microscopy characterization of two unit cells STO on SRO buffered (001) STO.** (**a**) AFM image of the STO. (**b**) Out-of-plane PFM phase image after writing +/-5 V voltage on the white dashed rectangle area. (**c**) Out-of-plane PFM phase image after three times conductive tip-grounded scans. (**d**) Local out-of-plane SS-PFM amplitude (blue line) and phase (red filled circles) in 4 cycles. The inset of (**d**) shows the bias-on PFM hysteresis curves. The scale bar is 1 μm.

We have grown 2-u.c. BTO and STO on SRO buffered STO and measure their ferroelectric properties by PFM in a similar manner with that in the main text. Supplementary Figure 21 shows the growth of BTO and STO under the same conditions monitoring by the mean of RHEED technique. All films show excellent quality and atomic flat surface. The PFM results of BTO and STO are shown in Supplementary Figures 22 and 23, respectively. Just after +/- 5V voltage poling, the PFM image of 2-u.c. BTO film shows a weak phase contrast, as shown in Supplementary Figure 22b. The weak contrast almost vanishes after three times of conductive tip-grounded scans. Five unit cells BTO shows typical hysteresis curves in the inset of Supplementary Figure 22d, while 2-u.c. BTO film shows a very weak hysteresis curves. For 2-u.c. STO film, PFM image shows a faint phase contrast after electrical poling in Supplementary Figure 23b. This faint phase contrast completely disappears after the conductive tip-grounded scan, as shown in Supplementary Figure 23c. Similarly, it doesn’t show any bias-off hysteresis signal, despite the bias-on signal being perfect hysteresis loops. This means 2-u.c. STO film is non-ferroelectric.


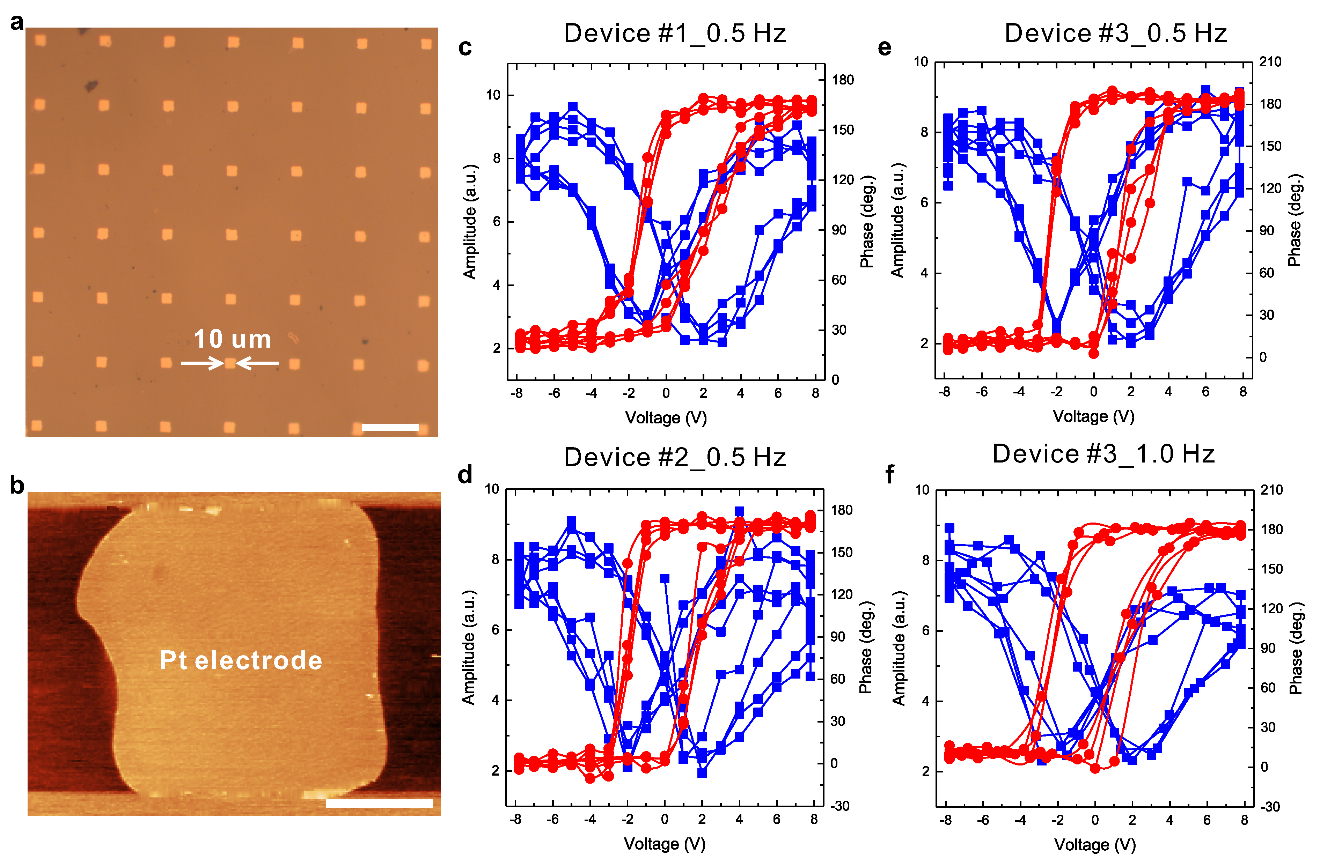


**Supplementary Figure 24 | Ferroelectric switching in two unit cells BFO with a square Pt top electrode.** (**a**) The optical image of device. The scale bar is 50 μm. (**b**) Topography of device by AFM. The scale bar is 5 μm. (**c**-**d**) Local hysteresis curves on different devices under IV_Frequency of 0.5 Hz. (**f**) Local hysteresis curves on the device (**e**) under IV_Frequency of 1 Hz.

To confirm the ferroelectricity in ultrathin BFO films, we try to measure the ferroelectric hysteresis loops directly on top electrode. 20 nm thick Pt film on BFO films is deposited by ultra-high vacuum magnet sputtering (Ar pressure: 5 mTorr, Power: 60 W). The device patterns (10*10 μm^2^) are fabricated by the lithography technique. Then the uncover portion is etched by ion milling. Finally, the photoresist is developed by acetone and DI water. The optical image of large-scale device pattern and topography of one device are shown in Supplementary Figure 24a and 24b. The hysteresis loops in 4 cycles of more than 10 device are measured in air by PFM technique, as shown in Supplementary Figure 24c- 24e. We also try to measure hysteresis loops under different IV-Frequency, as shown in Supplementary Figure 24f. The PFM phase loops show clear 180° switch behavior, which confirms that the polar state in ultrathin BFO could be repeatedly switched. The hysteresis loops under different IV_Frequency reveal that the switch behavior is stable.

**
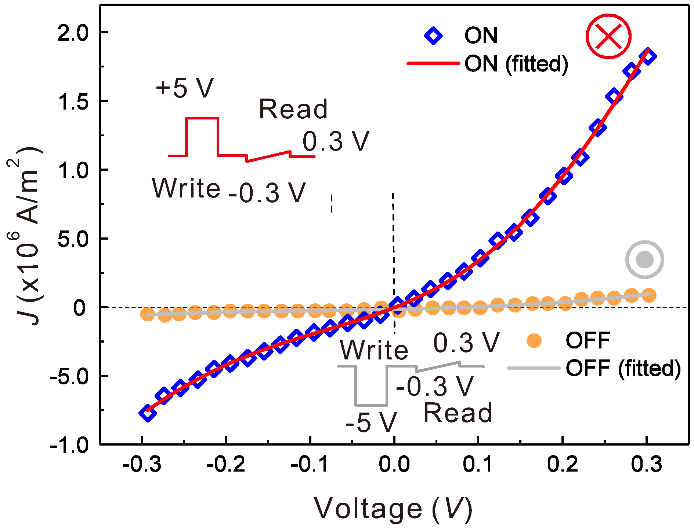
**

**Supplementary Figure 25 | TER effect in two unit cells of BFO.** *J*-*V* curves for two opposite polarization (blue, down, orange, up). Experimental data were fitted by pure direct tunneling mechanism based on the WKB theory. The inset illustrates the write and read voltage sequences for *J*-*V* measurements.

Supplementary Figure 25 shows the current density and voltage (*J*-*V*) behaviors in FTJ of 2-u.c. BFO. The *J*-*V* curves between -0.3 V and 0.3 V present two different states: low resistance state (ON) after negative writing and high resistance state (OFF) after positive writing. The *J*-*V* of ON state shows a parabolic relationship, according to with the direct quantum tunneling principle, while the curve of OFF state looks like an asymmetric Schottky-emission-type behavior. Both *J*-*V* curves are able to be well fitted by Wentzel-Kramers-Brillouin (WKB) approximation with the equation, as developed by Gruverman *et al*^31^. The maximum tunneling electroresistance (TER) ratio is as high as 2700%, which was attributed to a change in the potential barrier related to ferroelectric polarization switching.


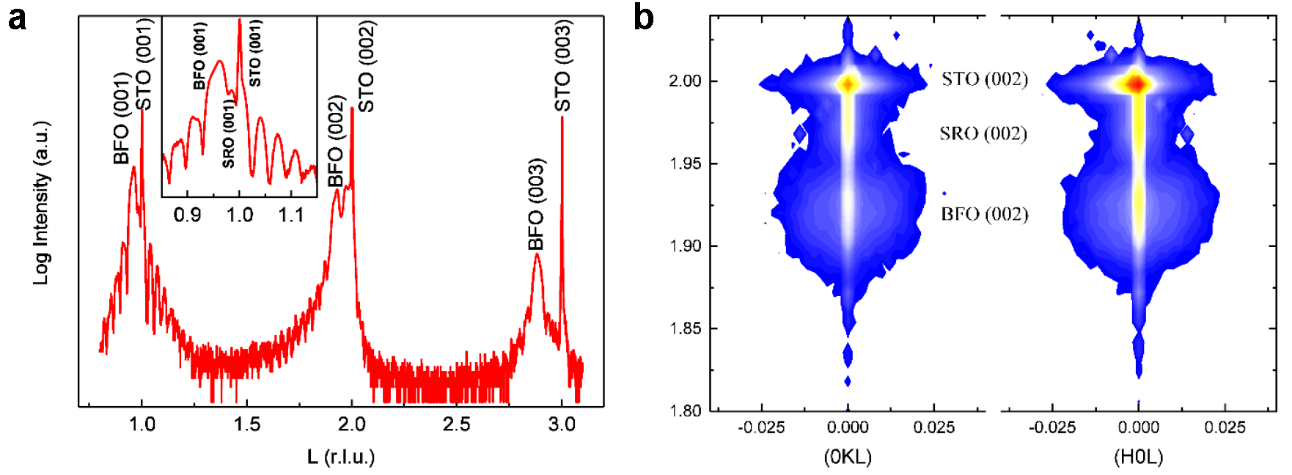


**Supplementary Figure 26 | High-resolution XRD results**. (**a**) The **L**-scan pattern of 12 nm BFO film deposited on SRO buffered STO. The inset shows the enlarged pattern at the (001) peak. (**b**) Symmetric RSM around {002}, left one has the incident X-ray parallel to [010] of STO, the right one is parallel to [100] of STO.

As shown in Supplementary Figure 26a, high-resolution X-ray diffraction (HR-XRD) **L**-scan displays only the 00*l* peaks from BFO, SRO films and STO substrate, indicating the coherently epitaxial growth of the films. The presence of Laue oscillations around the 00*l* peak reveals the perfect crystallinity and flat surface/interface. The symmetric RSM around 002 was shown in Supplementary Figure 26b. All of the BFO, SRO and STO peaks were located along the vertical line, indicating no tilt in [010] and [100] direction. The out-of-plane lattice constant *c*_pc_ is determined to be ∼3.9547 (or ~4.0622) Å according to the distance between the reciprocal points of SRO (or BFO) and that of STO.


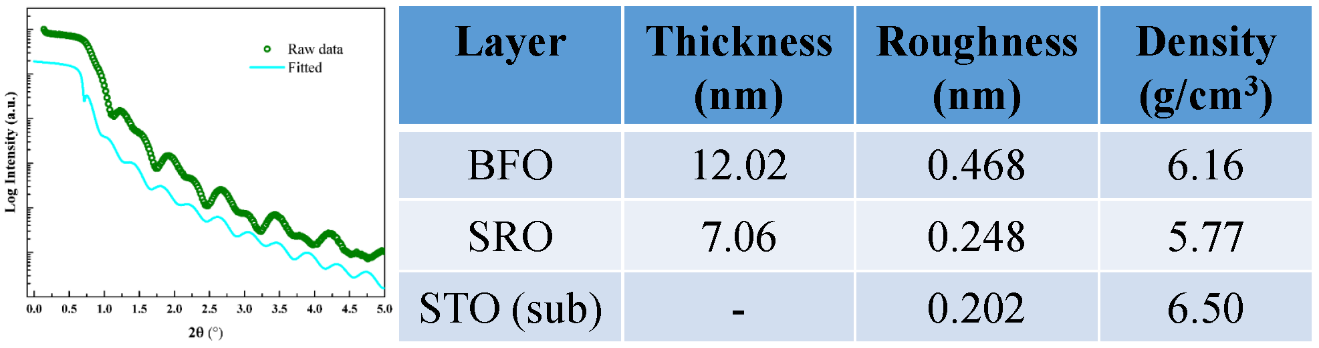


**Supplementary Figure 27 | XRR results of thick BFO film on SRO buffered STO**. Circle symbol represents the XRR results, while the solid line represents the fitted results. The film thickness, roughness at surface and interface and density of film and substrate determined by XRR fitted.

Supplementary Figure 27 shows the measured and fitted XRR curves for the thick BFO film. The well-resolved Kiessig fringes display two types of oscillations, which suggests two thickness and smooth surface/interface in BFO/SRO/STO heterostructure. The three-layer model including BFO, SRO films, and STO substrate is used to fit the XRR data. The layer thickness, surface/interface roughness and material density derived by fitting XRR result were listed in the Table. The thickness of SRO is consistent with the observation of STEM. The roughness of film and substrate are well consistent with the measurement of AFM.

**
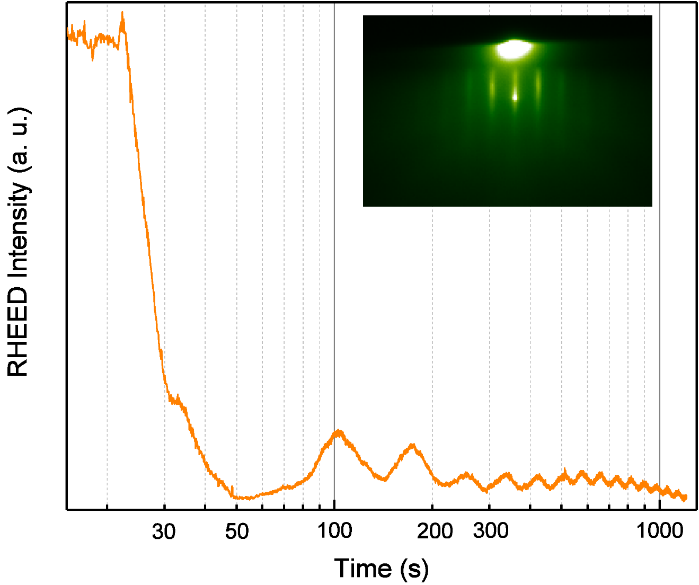
**

**Supplementary Figure 28 |** **Growth of thick BFO buffered by SRO on STO.** RHEED intensity oscillation monitoring for the growth of BFO**.** The inset shows RHEED patterns of the substrate after SRO and BFO deposition.

Supplementary Figure 28 shows the growth of 12 nm BFO buffered by SRO on STO substrate. The growth mechanism of BFO was monitored by RHEED. The first three oscillations display strong intensity changes, indicating a layer-by-layer growth mode, while the succeeding weaker oscillations indicate a step flow growth mode. Though the transition of growth mode from layer-by-layer to step flow appears, the RHEED pattern displays streaky lines, indicating the two-dimension surface of BFO film.


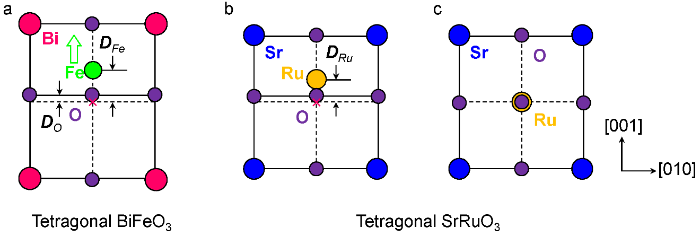


**Supplementary Figure 29 | Sketch of the atom displacement in tetragonal structure**. (**a**) Tetragonal BFO ferroelectric phase in a plane of (100). ***D****_Fe_* and ***D****_O_* denote the displacements of the Fe and O atoms along [001] direction from the positions of centrosymmetry. (**b**) Tetragonal SRO with the displacement of the Ru and O atoms along [001] direction. (**c**) Tetragonal centrosymmetric SRO.


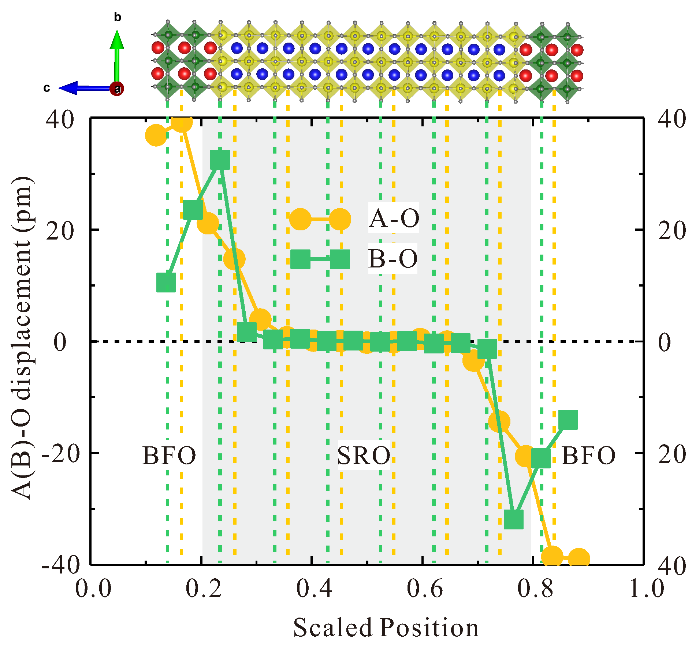


**Supplementary Figure 30 | Theoretical calculations indicating the off-center displacement**. Simulation cell containing BFO/SRO/BFO and 16-u.c. of vacuum (not shown). A-O and B-O displacement along the growth direction of the lattice. The middle SRO region is shaded. The in-plane lattice constant is fixed that (3.86 Å) of substrate SrTiO_3_. The vertical dashed lines help to guide the eyes.

In Supplementary Figure 30, we show a relaxed structure of BFO/SRO heterostructure with no rotation and tilt. Our structure includes RuO_2_ terminated SRO and BiO terminated BFO similar to the experimental structure. The A (Bi, Sr)-O and B (Fe, Ru)-O distance plotted in Supplementary Figure 30 supports the presence of significant polarization.

**Supplementary TABLE I | Cell parameters refined by RSV method for SRO and BFO deposited on STO**. Lattice parameters of Super-Tetragonal (Super-T) BFO and Rhombohedral (R) like BFO, Tetragonal (T) BTO and Tetragonal (T) PTO were listed for comparison. Parameters of *a*, *b* and *c* are the lattice constant along [100]_pc_, [010]_pc_ and [001]_pc_ axis, where PC represents pseudocubic. The error range of length is +/-0.001 Å, while the error range of angle is +/-0.002 º.

| (Å/°) | SRO  (Our work) | T-BFO  (Our work) | Super-T BFO  (Ref. ^32^) | R-BFO  (Ref. ^33^) | T-BTO  (Ref. ^34^) | T-PTO  (Ref. ^35^) |
| --- | --- | --- | --- | --- | --- | --- |
| a | 3.9056 | 3.9061 | 3.818 | 3.907 | 4.00 | 3.904 |
| b | 3.9051 | 3.9057 | 3.740 | 3.973 | 4.00 | 3.904 |
| c | 3.9547 | 4.0622 | 4.662 | 3.997 | 4.10 | 4.125 |
|  | 90.005 | 90.015 | 90.00 | 90.00 | 90.00 | 90.00 |
|  | 89.998 | 89.952 | 88.12 | 89.2 | 90.00 | 90.00 |
|  | 90.008 | 90.000 | 90.00 | 90.00 | 90.00 | 90.00 |
| c/a | 1.01 | 1.04 | 1.22 | 1.02 | 1.03 | 1.06 |

Consequently, accurate lattice parameters can be refined from the position of reciprocal lattice point of films in reciprocal space of substrate by RSV method^1^, listed in Supplementary Table I.

**Supplementary TABLE II | Comparison of TER performance of FTJ with an ultrathin ferroelectric layer.** *V*_R_ represents read voltage. Size represents the diameter of the device. SPM is scanning probe microscopy method. The term of NSTO represents Nb-doped SrTiO_3_.

| Device | TER (%) | *V*_R_ (V) | Size (nm) | Method | Ref. |
| --- | --- | --- | --- | --- | --- |
| (2 u.c.) BTO/STO_SRO_ | 140 | -1.1 | 500 | 2-Probe | ^36^ |
| (6 u.c.) BTO_SRO_ | 700 | 0.3 | 20 | SPM | ^31^ |
| (2 u.c.) BTO_NSTO_ | 400 | 2.0 | 5*10^3^ | 2-Probe | ^37^ |
| (2 u.c.) BTO_LSMO_ | 200 | 1.5 | 20 | SPM | ^17^ |
| (8 u.c.) PZT_LSMO_ | 300 | -1.5 | 20 | SPM | ^38^ |
| (5 u.c.) LBMO_LSMO_ | 5 | 0.01 | 40 | 2-Probe | ^20^ |
| 1 layer PVDF (2.2 nm) | 300 | <0.5 | 190 | SPM | ^39^ |
| 2 layer PVDF (4.5 nm) | 1000 | <0.5 | 190 | SPM | ^39^ |
| (1 u.c.) T-BFO_SRO_ | 370 | 0.3 | 25 | SPM | Our work |
| (2 u.c.) T-BFO_SRO_ | 2700 | 0.3 | 25 | SPM | Our work |

We compare the present BFO-FTJ with other similar ultrathin FTJ reported recently, listed in Supplementary Table II. It can be seen that the T-BFO devices display higher TER performance at a smaller thickness, which is very crucial for the application in miniaturized electronic devices.

**Supplementary References:**

1 Yang, P., Liu, H., Chen, Z., Chen, L. & Wang, J. Unit-cell determination of epitaxial thin films based on reciprocal-space vectors by high-resolution X-ray diffractometry. *J. Appl. Crystallogr.* **47**, 402 (2014).

2 Graf, M., Sepliarsky, M. & Stachiotti, M. G. Atomic-level study of BiFeO_3_ under epitaxial strain. *Phys. Rev. B* **94**, 054101 (2016).

3 Zhang, J. X. *et al.* Microscopic Origin of the Giant Ferroelectric Polarization in Tetragonal-like BiFeO_3_. *Phys. Rev. Lett.* **107**, 147602 (2011).

4 Ravindran, P., Vidya, R., Kjekshus, A., Fjellvåg, H. & Eriksson, O. Theoretical investigation of magnetoelectric behavior in BiFeO_3_. *Phys. Rev. B* **74**, 224412 (2006).

5 Nelson, C. T. *et al.* Spontaneous vortex nanodomain arrays at ferroelectric heterointerfaces. *Nano Lett.* **11**, 828 (2011).

6 Tang, Y. L. *et al.* Observation of a periodic array of flux-closure quadrants in strained ferroelectric PbTiO_3_ films. *Science* **348**, 547 (2015).

7 Beekman, C. *et al.* Ferroelectric Self-Poling, Switching, and Monoclinic Domain Configuration in BiFeO_3_ Thin Films. *Adv. Funct. Mater.* **26**, 5166 (2016).

8 Lubk, A., Gemming, S. & Spaldin, N. A. First-principles study of ferroelectric domain walls in multiferroic bismuth ferrite. *Phys. Rev. B* **80**, 104110 (2009).

9 Catalan, G. & Scott, J. F. Physics and Applications of Bismuth Ferrite. *Adv. Mater.* **21**, 2463 (2009).

10 Kresse, G. & Furthmüller, J. Efficient iterative schemes forab initiototal-energy calculations using a plane-wave basis set. *Phys. Rev. B* **54**, 11169 (1996).

11 Kresse, G. & Joubert, D. From ultrasoft pseudopotentials to the projector augmented-wave method. *Phys. Rev. B* **59**, 1758 (1999).

12 Blöchl, P. E. Projector augmented-wave method. *Phys. Rev. B* **50**, 17953 (1994).

13 Monkhorst, H. J. & Pack, J. D. Special points for Brillouin-zone integrations. *Phys. Rev. B* **13**, 5188 (1976).

14 Paudel, T. R., Jaswal, S. S. & Tsymbal, E. Y. Intrinsic defects in multiferroic BiFeO_3_ and their effect on magnetism. *Phys. Rev. B* **85**, 104409 (2012).

15 Dudarev, S. L., Botton, G. A., Savrasov, S. Y., Humphreys, C. J. & Sutton, A. P. Electron-energy-loss spectra and the structural stability of nickel oxide:An LSDA+U study. *Phys. Rev. B* **57**, 1505 (1998).

16 Bark, C. W. *et al.* Switchable induced polarization in LaAlO_3_/SrTiO_3_ heterostructures. *Nano Lett.* **12**, 1765 (2012).

17 Garcia, V. *et al.* Giant tunnel electroresistance for non-destructive readout of ferroelectric states. *Nature* **460**, 81 (2009).

18 Yamada, H. *et al.* Strong Surface-Termination Effect on Electroresistance in Ferroelectric Tunnel Junctions. *Adv. Funct. Mater.* **25**, 2708 (2015).

19 Crassous, A. *et al.* Giant tunnel electroresistance with PbTiO_3_ ferroelectric tunnel barriers. *Appl. Phys. Lett.* **96** (2010).

20 Gajek, M. *et al.* Tunnel junctions with multiferroic barriers. *Nat. Mater.* **6**, 296 (2007).

21 Barrionuevo, D. *et al.* Tunneling electroresistance in multiferroic heterostructures. *Nanotechnology* **25**, 495203 (2014).

22 Hu, W. J., Wang, Z., Yu, W. & Wu, T. Optically controlled electroresistance and electrically controlled photovoltage in ferroelectric tunnel junctions. *Nat. Commun.* **7**, 10808 (2016).

23 Lee, K. Y. *et al.* Controllable Charge Transfer by Ferroelectric Polarization Mediated Triboelectricity. *Adv. Funct. Mater.* **26**, 3067 (2016).

24 Kim, Y. *et al.* Origin of surface potential change during ferroelectric switching in epitaxial PbTiO_3_ thin films studied by scanning force microscopy. *Appl. Phys. Lett.* **94**, 032907 (2009).

25 Balke, N. *et al.* Exploring local electrostatic effects with scanning probe microscopy: implications for piezoresponse force microscopy and triboelectricity. *ACS Nano* **8**, 10229 (2014).

26 Balke, N. *et al.* Differentiating Ferroelectric and Nonferroelectric Electromechanical Effects with Scanning Probe Microscopy. *ACS Nano* **9**, 6484 (2015).

27 Vasudevan, R. K., Balke, N., Maksymovych, P., Jesse, S. & Kalinin, S. V. Ferroelectric or non-ferroelectric: Why so many materials exhibit “ferroelectricity” on the nanoscale. *App. Phys. Rev.* **4**, 021302 (2017).

28 Vasudevan, R. K. *et al.* Exploring topological defects in epitaxial BiFeO_3_ thin films. *ACS Nano* **5**, 879 (2011).

29 Xi, Z. *et al.* Giant tunnelling electroresistance in metal/ferroelectric/semiconductor tunnel junctions by engineering the Schottky barrier. *Nat. Commun.* **8**, 15217 (2017).

30 Lu, W., Yang, P., Song, W. D., Chow, G. M. & Chen, J. S. Control of oxygen octahedral rotations and physical properties in SrRuO_3_ films. *Phys. Rev. B* **88**, 214115 (2013).

31 Gruverman, A. *et al.* Tunneling electroresistance effect in ferroelectric tunnel junctions at the nanoscale. *Nano Lett.* **9**, 3539 (2009).

32 Chen, Z. *et al.* Low-Symmetry Monoclinic Phases and Polarization Rotation Path Mediated by Epitaxial Strain in Multiferroic BiFeO_3_ Thin Films. *Adv. Funct. Mater.* **21**, 133 (2011).

33 Xu, G. *et al.* Low symmetry phase in (001) BiFeO_3_ epitaxial constrained thin films. *Appl. Phys. Lett.* **86**, 182905 (2005).

34 Trithaveesak, O., Schubert, J. & Buchal, C. Ferroelectric properties of epitaxial BaTiO_3_ thin films and heterostructures on different substrates. *J. Appl. Phys.* **98**, 114101 (2005).

35 Sun, L. *et al.* X-ray analysis of epitaxial thin film grown on an (001) substrate by metal-organic chemical vapour deposition. *J. Phys. Condens. Matter* **8**, 10185 (1996).

36 Wang, L. *et al.* Overcoming the Fundamental Barrier Thickness Limits of Ferroelectric Tunnel Junctions through BaTiO_3_/SrTiO_3_ Composite Barriers. *Nano Lett.* **16**, 3911 (2016).

37 Li, C. *et al.* Ultrathin BaTiO_3_-Based Ferroelectric Tunnel Junctions through Interface Engineering. *Nano Lett.* **15**, 2568 (2015).

38 Jiang, L. *et al.* Tunneling electroresistance induced by interfacial phase transitions in ultrathin oxide heterostructures. *Nano Lett.* **13**, 5837 (2013).

39 Tian, B. B. *et al.* Tunnel electroresistance through organic ferroelectrics. *Nat. Commun.* **7**, 11502 (2016).
